# The signal peptide of Cry1Ia can improve the expression of eGFP or mCherry in *Escherichia coli* and *Bacillus thuringiensis* and enhance the host’s fluorescent intensity

**DOI:** 10.1186/s12934-020-01371-8

**Published:** 2020-05-24

**Authors:** Jianhua Gao, Hongmei Qian, Xiaoqin Guo, Yi Mi, Junpei Guo, Juanli Zhao, Chao Xu, Ting Zheng, Ming Duan, Zhongwei Tang, Chaoyang Lin, Zhicheng Shen, Yiwei Jiang, Xingchun Wang

**Affiliations:** 1 College of Life Sciences, Shanxi Agricultural University, Taigu, 030801 China; 2 State Key Laboratory of Rice Biology, Institute of Insect Sciences, College of Agriculture and Biotechnology, Zhejiang University, Hangzhou, 310058 China; 3 Experimental Teaching Center, Shanxi Agricultural University, Taigu, 030801 China; 4 Department of Agronomy, Purdue University, West Lafayette, IN 47907 USA

**Keywords:** Signal peptide, Cry1Ia, Fusion tag, Fluorescent proteins, Fluorescent intensity, Expression level

## Abstract

**Background:**

The signal peptides (SPs) of secretory proteins are frequently used or modified to guide recombinant proteins outside the cytoplasm of prokaryotic cells. In the periplasmic space and extracellular environment, recombinant proteins are kept away from the intracellular proteases and often they can fold correctly and efficiently. Consequently, expression levels of the recombinant protein can be enhanced by the presence of a SP. However, little attention has been paid to the use of SPs with low translocation efficiency for recombinant protein production. In this paper, the function of the signal peptide of *Bacillus thuringiensis* (Bt) Cry1Ia toxin (Iasp), which is speculated to be a weak translocation signal, on regulation of protein expression was investigated using fluorescent proteins as reporters.

**Results:**

When fused to the N-terminal of eGFP or mCherry, the Iasp can improve the expression of the fluorescent proteins and as a consequence enhance the fluorescent intensity of both *Escherichia coli* and Bt host cells. Real-time quantitative PCR analysis revealed the higher transcript levels of *Iegfp* over those of *egfp* gene in *E. coli* TG1 cells. By immunoblot analysis and confocal microscope observation, lower translocation efficiency of IeGFP was demonstrated. The novel fluorescent fusion protein IeGFP was then used to compare the relative strengths of *cry1Ia* (*P*_*i*_) and *cry1Ac* (*P*_*ac*_) gene promoters in Bt strain, the latter promoter proving the stronger. The eGFP reporter, by contrast, cannot indicate unambiguously the regulation pattern of *P*_*i*_ at the same level of sensitivity. The fluorescent signals of *E. coli* and Bt cells expressing the Iasp fused mCherry (ImCherry) were also enhanced. Importantly, the Iasp can also enhanced the expression of two difficult-to-express proteins, matrix metalloprotease-13 (MMP13) and myostatin (growth differentiating factor-8, GDF8) in *E. coli* BL21-star (DE3) strain.

**Conclusions:**

We identified the positive effects of a weak signal peptide, Iasp, on the expression of fluorescent proteins and other recombinant proteins in bacteria. The produced IeGFP and ImCherry can be used as novel fluorescent protein variants in prokaryotic cells. The results suggested the potential application of Iasp as a novel fusion tag for improving the recombinant protein expression.

## Introduction

GFP is the first fluorescent protein (FP) discovered in *Aequorea victoria* and has been widely used as a molecular reporter (reviewed in [1–3]). However, in bacteria expression systems, such as *Escherichia coli*, GFP cannot fluoresce efficiently due to poor three-dimensional (3D) structure formation [4–6]. When fusing with other proteins of interest, GFP may even interfere with the folding of its partner in bacteria [7]. The phenomenon was attributed to the high contact order topology of GFP and the lack of more advanced co-translational folding mechanisms in bacteria [7, 8]. Although the chromophore of GFP contains only three contiguous amino acids (Ser65-Tyr66-Gly67), its formation depends on the native β-barrel structure of the whole molecule [1, 9]. The final β-barrel structure of GFP needs contacts among distant residues in the primary sequence, leading to the low folding rates. Thus, when highly expressed in prokaryotic cells, the GFP peptides aggregate in the crowded cytosol before they can correctly fold.

Strategies have been developed to improve the folding efficiency and fluorescing of GFP in bacteria. Point mutations were showed to improve the fluorescence and/or the folding of GFP. Out of these reported mutants, the enhanced GFP (eGFP) [10] and the superfolder GFP (sfGFP) [11] are widely used. Besides the modification of the GFP molecule per se, general methods to improve the expression of recombinant proteins have also been used [12–17]. For instance, the productive folding of N-terminal partner in fusion proteins with GFP could prevent the aggregation of the whole proteins [18]. The popular solubility enhancer partners maltose-binding protein (MBP) and N-utilization substance (NusA) can significantly enhance the solubility of GFP at low temperature [19], but the positive effects disappeared at high temperature [7]. Recently, several novel fusion tags, including Fh8 (an 8-kDa calcium-binding protein extracted from *Fasciola hepatica*), LEA-like peptide (a hydrophilic late embryogenesis abundant peptide), SKIK (Ser-Lys-Ile-Lys) peptide, *Os*TDX (*Oryza sativa* tetratricopeptide domain-containing thioredoxin), S1v1 (a self-assembling amphipathic peptide) and NT11 (the first 11 amino acid residues of a duplicated carbonic anhydrase), were reported can improve the expression of fluorescent proteins in bacteria [17, 20–24]. Chaperones, such as the group containing the DnaK and co-chaperones DnaJ and GrpE (K/J/E), can help the folding of the corresponding aggregation-prone partner and sequentially enhance the fluorescence of GFP in fusion protein [25]. A newly identified molecular chaperone Spheroplast Protein Y (Spy) can be used as a remarkable solubility enhancing fusion tag and its tandem pattern enable it to enhance the soluble expression of large-size proteins [26]. Codon usage and mRNAstructure also affect the fluorescing of GFP [27]. Siller et al. [28] used mutant ribosomes to reduce polypeptide elongation rates, thus facilitated the folding efficiency of recombinant proteins, especially from eukaryotes. This generalized reduction strategy did not adversely impact the folding of the endogenous bacterial proteome.

Generally, the signal peptides (SPs) or their mutants are used to guide the recombinant proteins into periplasmic space or out of the cell for improving the structure formation or the expression level (reviewed in [12–14]). But the GFP protein cannot fold into its fluorescent form upon translocation via the SecYEG-translocon due to the post translocation folding mechanism [15, 29, 30]. Unlike the SecYEG-translocon, the twin-arginine translocation system (Tat system) only guides the fully folded and/or co-factor incorporated proteins translocation [31, 32]. As expected, the fluorescent signal of GFP molecules translocated into periplasmic space by the signal peptide of torA (trimethylamine N-oxide reductase) was observed [33]. The signal peptide of torA was also reported to be an inclusion body tag that can aggregate the recombinant proteins in *E. coli* cytosol [34]. It was not only a novel application of SPs, but also prompted us to pay more attention to the diversity of SPs. In this study, we used a novel fusion tag, the signal peptide of Cry1Ia (Iasp), to improve the expression of GFP in *E. coli* and *Bacillus thuringiensis* (Bt).

Cry1I toxins of Bt belong to a special branch of the Cry protein family (reviewed in [35, 36]). The *cry1I* genes generally locate at approximately 500 bp downstream of the *cry1A* genes. Since there is not a classic promoter structure, the regulation of *cry1I* genes was a mystery until their transcripts were identified by Tounsi and Jaoua [37–39]. Another special characteristic of Cry1I protein is the loss of the classic long C-terminal peptide of Cry1 toxins, which plays an important role in folding the N-terminal three domains into parasporal crystal. The absence of the C-terminal part in Cry1I proteins suggested a different expression pattern. In fact, Kostichka et al. [40] identified Cry1Ia4 protein in supernatant fluids of Bt AB88 cultures and predicted its secretion signal peptide (N-terminal 45 amino acids) which can be removed by unknown peptidase(s) after translocation. Although the Cry1Ia was showed to be a secretory protein, some of the expressed products were observed in the cell pellets of the natural host. The results suggested that the translocation capacity of the signal peptide of Cry1Ia was not high or the corresponding secretory pathway was easily saturated by the overexpressed targets [41, 42]. At present, the related secretory pathway of Cry1Ia is still unclear, but it could be excluded from the Tat system due to lack of the classic conversed sequence (S/T-RRXFLK), especially the double-arginine motif, in signal sequence [43–45].

The effects of the Iasp on the eGFP and mCherry proteins in *E. coli* and Bt cells were evaluated. The results showed that the N-terminal location of Iasp in the fusion fluorescent proteins improved the expression of eGFP and mCherry and consequently enhanced the fluorescent intensity of host cells. The produced IeGFP and ImCherry can be used as ideal reporters in prokaryotic cells. Moreover, the production yields of other difficult-to-express recombinant proteins, such as matrix metalloprotease-13 (MMP13) and myostatin (growth differentiating factor-8, GDF8) in *E. coli* BL21-star (DE3) strain [19], were dramatically enhanced after fusing with the Iasp. These results indicated that the Iasp can be used as a novel fusion tag for enhancing recombinant protein expression.

## Results

### Fluorescent intensity detection of IeGFP in *E. coli*

**Fig. 1:**
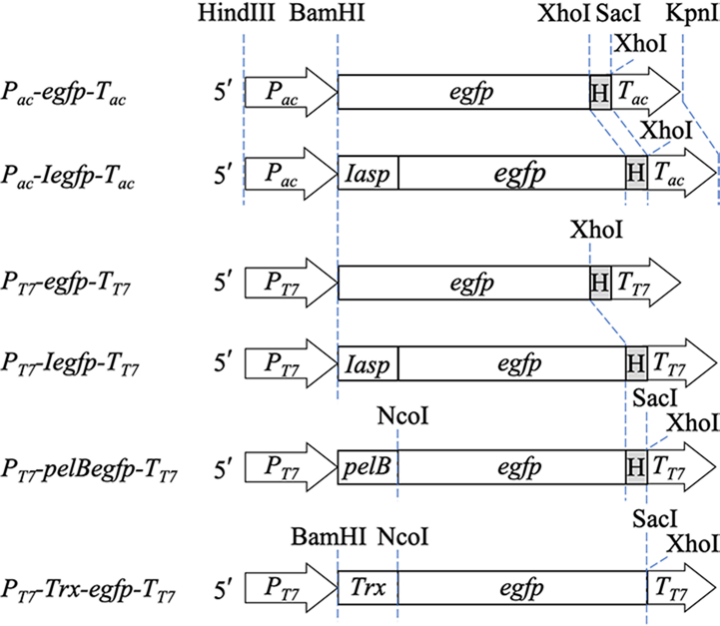
The expression cassettes of the fusion fluorescent proteins. The *P*_*ac*_-*pelBegfp*-*T*_*ac*_ and *P*_*ac*_-*torAegfp*-*T*_*ac*_ expression cassettes are similar to the *P*_*ac*_-*Iegfp*-*T*_*ac*_ except the leader sequence at the 5′ flanking of *egfp* gene. The expression cassettes *P*_*T7*_-*NusA*-*egfp*-*T*_*T7*_, *P*_*T7*_-*MBP*-*egfp*-*T*_*T7*_, *P*_*T7*_-*I*-*mmp13*-*T*_*T7*_, *P*_*T7*_-*Trx*-*mmp13*-*T*_*T7*_, *P*_*T7*_-*I*-*gdf8*-*T*_*T7*_ and *P*_*T7*_-*Trx*-*gdf8*-*T*_*T7*_ share same structures with *P*_*T7*_-*Trx*-*egfp*-*T*_*T7*_

**Fig. 2:**
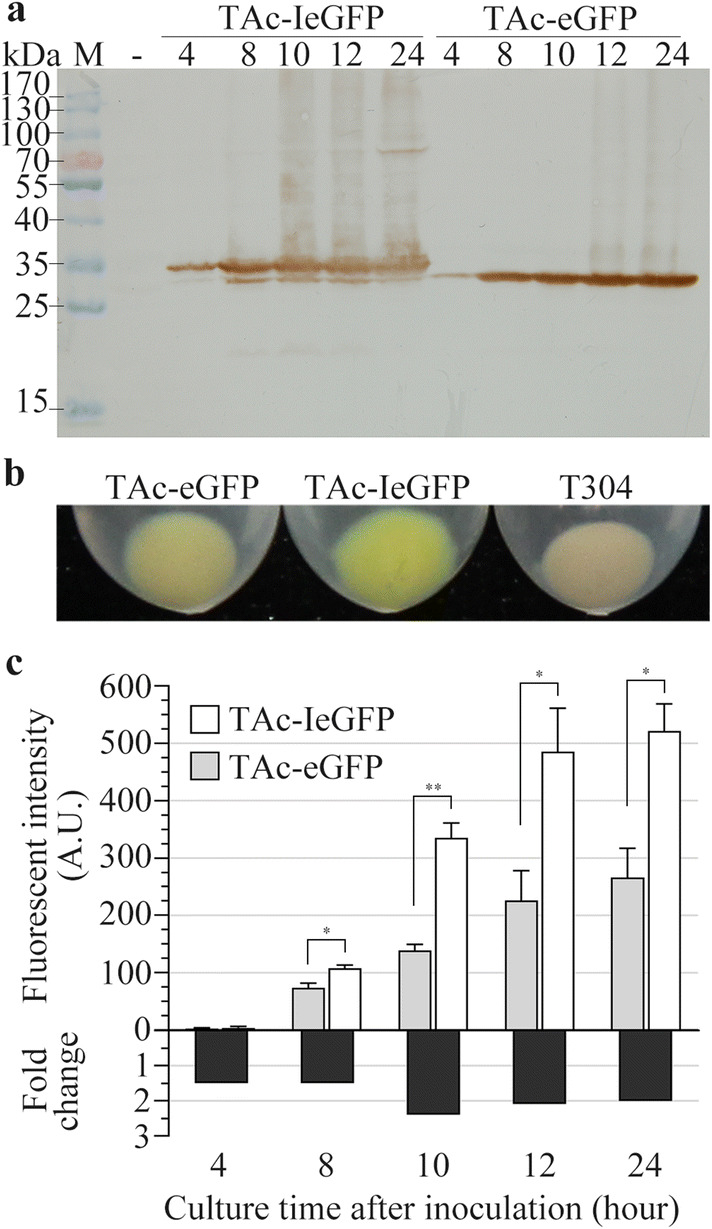
Expression analysis of eGFP and IeGFP regulated by *P*_*ac*_ promoter in *E.coli* TG1 strain. **a** Lane “-” is the negative control which prepared from T304 cells sampled at 12 h after inoculation. TAc-IeGFP and TAc-eGFP cells were separately taken at 4, 8, 10, 12 and 24 h after inoculation. Lane “M” is the molecular weight standards. **b** Comparison of the collected cells when they were incubated for 24 h. **c** The fluorescent intensities and their fold changes for TAc-IeGFP over TAc-eGFP strains at corresponding sampling time. The slit widths of EX/EM were 3 nm and 5 nm respectively and the detections were conducted in low sensitivity. The fluorescent signals of T304 cells cannot be detected. The error bars indicate standard error of mean. The significant differences of the fluorescent intensities between TAc-IeGFP and TAc-eGFP cells at corresponding time were indicated by single asterisk (p < 0.05) or double asterisks (p < 0.01)

The expression cassettes and the corresponding plasmids and strains used in this study were showed in Fig. 1 and Table 1. As a constitutive promoter in *E. coli*, the promoter of *cry1Ac* gene (*P*_*ac*_) can regulate the expression of its downstream gene uninterruptedly after inoculation [46]. As expected, the expression of both IeGFP and eGFP was detected at 4 h after inoculation by immunoblot analysis (Fig. 2a). The expression products cannot be identified easily by Coomassie bright blue staining SDS-PAGE analysis (data not shown). The migration of intact IeGFP proteins was consistent with the calculated molecular weight (33.1 kDa). Interestingly, only a small quantity of IeGFP proteins were cleaved to give the same molecular weight product (27.9 kDa) as eGFP. The more intense color of cell pellets of strain TAc-IeGFP clearly distinguished them from pellets of strain TAc-eGFP (Fig. 2b).

**Table 1:** Plasmids and strains used and constructed in this work

Plasmid	Characteristics	Source
pHT304	*Amp*^*r*^, *Erm*^*r*^, *Bacillus*-*E. coli* shuttle vector	[64]
p304ΔSacI	pHT304 without SacI recognition site	This work
pAc-IeGFP	p304ΔSacI, *P*_*ac*_-*Iegfp*-*T*_*ac*_ expression cassette	This work
pAc-eGFP	p304ΔSacI, *P*_*ac*_-*egfp*-*T*_*ac*_ expression cassette	This work
pAc-pelBeGFP	p304ΔSacI, *P*_*ac*_-*pelBegfp*-*T*_*ac*_ expression cassette	This work
pAc-torAeGFP	p304ΔSacI, *P*_*ac*_-*torAegfp*-*T*_*ac*_ expression cassette	This work
pAc-eGFPI	p304ΔSacI, *P*_*ac*_-*egfpI*-*T*_*ac*_ expression cassette	This work
pI-IeGFP	p304ΔSacI, *P*_*i*_-*Iegfp*-*T*_*ac*_ expression cassette	This work
pI-eGFP	p304ΔSacI, *P*_*i*_-*egfp*-*T*_*ac*_ expression cassette	This work
pAc-ImCherry	p304ΔSacI, *P*_*ac*_-*ImCherry*-*T*_*ac*_ expression cassette	This work
pAc-mCherry	p304ΔSacI, *P*_*ac*_-*mCherry*-*T*_*ac*_ expression cassette	This work
pET28aDel	*Kan*^*r*^, pET28a without the sequence between RBS and BamHI	[61]
pET28aDel2	*Kan*^*r*^, add the GATATA at the 5′ of BamHI site of pET28aDel	This work
p28aD-IeGFP	pET28aDel, *P*_*T7*_-*Iegfp*-*T*_*T7*_ expression cassette	This work
p28aD-eGFP	pET28aDel, *P*_*T7*_-*egfp*-*T*_*T7*_ expression cassette	This work
p22b-eGFP	pET22b, *P*_*T7*_-*pelBegfp*-*T*_*T7*_ expression cassette	This work
pCMV-N-mCherry	*mCherry* gene	Beyotime biotechnology, China
p28aD2-T-eGFP	pET28aDel2, *P*_*T7*_-*Trx*-*egfp*-*T*_*T7*_ expression cassette	This work
p28aD2-N-eGFP	pET28aDel2, *P*_*T7*_-*NusA*-*egfp*-*T*_*T7*_ expression cassette	This work
p28aD2-M-eGFP	pET28aDel2, *P*_*T7*_-*MBP*-*egfp*-*T*_*T7*_ expression cassette	This work
p28aD2-MMP13	pET28aDel2, *P*_*T7*_-*mmp13*-*T*_*T7*_ expression cassette	This work
p28aD2-GDF8	pET28aDel2, *P*_*T7*_-*gdf8*-*T*_*T7*_ expression cassette	This work
p28aD2-I-MMP13	pET28aDel2, *P*_*T7*_-*I*-*mmp13*-*T*_*T7*_ expression cassette	This work
p28aD2-T-MMP13	pET28aDel2, *P*_*T7*_-*Trx*-*mmp13*-*T*_*T7*_ expression cassette	This work
p28aD2-I-GDF8	pET28aDel2, *P*_*T7*_-*I*-*gdf8*-*T*_*T7*_ expression cassette	This work
p28aD2-T-GDF8	pET28aDel2, *P*_*T7*_-*Trx*-*gdf8*-*T*_*T7*_ expression cassette	This work
p28aD-Cry1Ia	pET28aDel, *P*_*T7*_-*cry1Ia*-*T*_*T7*_ expression cassette	This work
p28aD-Cry1IaD44	pET28aDel, *P*_*T7*_-*cry1IaD44*-*T*_*T7*_ expression cassette	This work
p28aD-Cry1IaD73	pET28aDel, *P*_*T7*_- *cry1IaD73*-*T*_*T7*_ expression cassette	This work
pAc-Cry1Ia	p304ΔSacI, *P*_*ac*_-*cry1Ia*-*T*_*ac*_ expression cassette	This work
pAc-Cry1IaD44	p304ΔSacI, *P*_*ac*_-*cry1IaD44*-*T*_*ac*_ expression cassette	This work
pAc-Cry1IaD73	p304ΔSacI, *P*_*ac*_-*cry1IaD73*-*T*_*ac*_ expression cassette	This work

Strain	Genotype	
*E. coli*		
TG1	*K12Δ(lac*-*pro), supE, thi, hsdD5,* F^–^ [*traD36 proAB*^+^*lacI*^q^*lacZΔM15*]	[65]
BL21-star (DE3)	F^–^*omp*T *hsd*S_B_ (r_B_^−^, m_B_^−^) *galdcmrne*131 (DE3)	Invitrogen
T304	TG1 transformed with pHT304	This work
TAc-IeGFP	TG1 transformed with pAc-IeGFP	This work
TAc-eGFP	TG1 transformed with pAc-eGFP	This work
TAc-pelBeGFP	TG1 transformed with pAc-pelBeGFP	This work
TAc-torAeGFP	TG1 transformed with pAc-torAeGFP	This work
TAc-eGFPI	TG1 transformed with pAc-eGFPI	This work
TAc-ImCherry	TG1 transformed with pAc-ImCherry	This work
TAc-mCherry	TG1 transformed with pAc-mCherry	This work
BL28aD	BL21-star (DE3) transformed with pET28aDel	This work
BL28-IeGFP	BL21-star (DE3) transformed with p28aD-IeGFP	This work
BL28-eGFP	BL21-star (DE3) transformed with p28aD-eGFP	This work
BL22b	BL21-star (DE3) transformed with pET22b	This work
BL22-eGFP	BL21-star (DE3) transformed with p22b-eGFP	This work
BL28D2-T-eGFP	BL21-star (DE3) transformed with p28aD2-T-eGFP	This work
BL28D2-N-eGFP	BL21-star (DE3) transformed with p28aD2-N-eGFP	This work
BL28D2-M-eGFP	BL21-star (DE3) transformed with p28aD2-M-eGFP	This work
BL28D2-MMP13	BL21-star (DE3) transformed with p28aD2-MMP13	This work
BL28D2-GDF8	BL21-star (DE3) transformed with p28aD2-GDF8	This work
BL28D2-I-MMP13	BL21-star (DE3) transformed with p28aD2-I-MMP13	This work
BL28D2-T-MMP13	BL21-star (DE3) transformed with p28aD2-T-MMP13	This work
BL28D2-I-GDF8	BL21-star (DE3) transformed with p28aD2-I-GDF8	This work
BL28D2-T-GDF8	BL21-star (DE3) transformed with p28aD2-T-GDF8	This work
BL28-Cry1Ia	BL21-star (DE3) transformed with p28aD-Cry1Ia	This work
BL28-Cry1IaD44	BL21-star (DE3) transformed with p28aD-Cry1Ia44	This work
BL28-Cry1IaD73	BL21-star (DE3) transformed with p28aD-Cry1Ia73	This work
*B. thuringiensis*		
BMB171	acrystalliferous mutant strain of YBT-1463	[66]
B304	BMB171 transformed with pHT304	This work
BAc-IeGFP	BMB171 transformed with pAc-IeGFP	This work
BAc-eGFP	BMB171 transformed with pAc-eGFP	This work
BI-IeGFP	BMB171 transformed with pI-IeGFP	This work
BI-eGFP	BMB171 transformed with pI-eGFP	This work
BAc-ImCherry	BMB171 transformed with pAc-ImCherry	This work
BAc-mCherry	BMB171 transformed with pAc-mCherry	This work
BAc-Cry1Ia	BMB171 transformed with pAc-Cry1Ia	This work
BAc-Cry1IaD44	BMB171 transformed with pAc-Cry1IaD44	This work
BAc-Cry1IaD73	BMB171 transformed with pAc-Cry1IaD73	This work

The fluorescent signals of TAc-IeGFP and TAc-eGFP strains were also monitored. An increasing trend of the fluorescent intensities for both TAc-IeGFP and TAc-eGFP was observed over time (Fig. 2c and Additional file 1: Table S1). Importantly, the fluorescent intensities of TAc-IeGFP strain were approximately 1.5, 2.4, 2.1 or 2.0-fold higher than TAc-eGFP strain at 8, 10, 12 or 24 h after inoculation, respectively.

### Fluorescent intensity detection of IeGFP in Bt strain

**Fig. 3:**
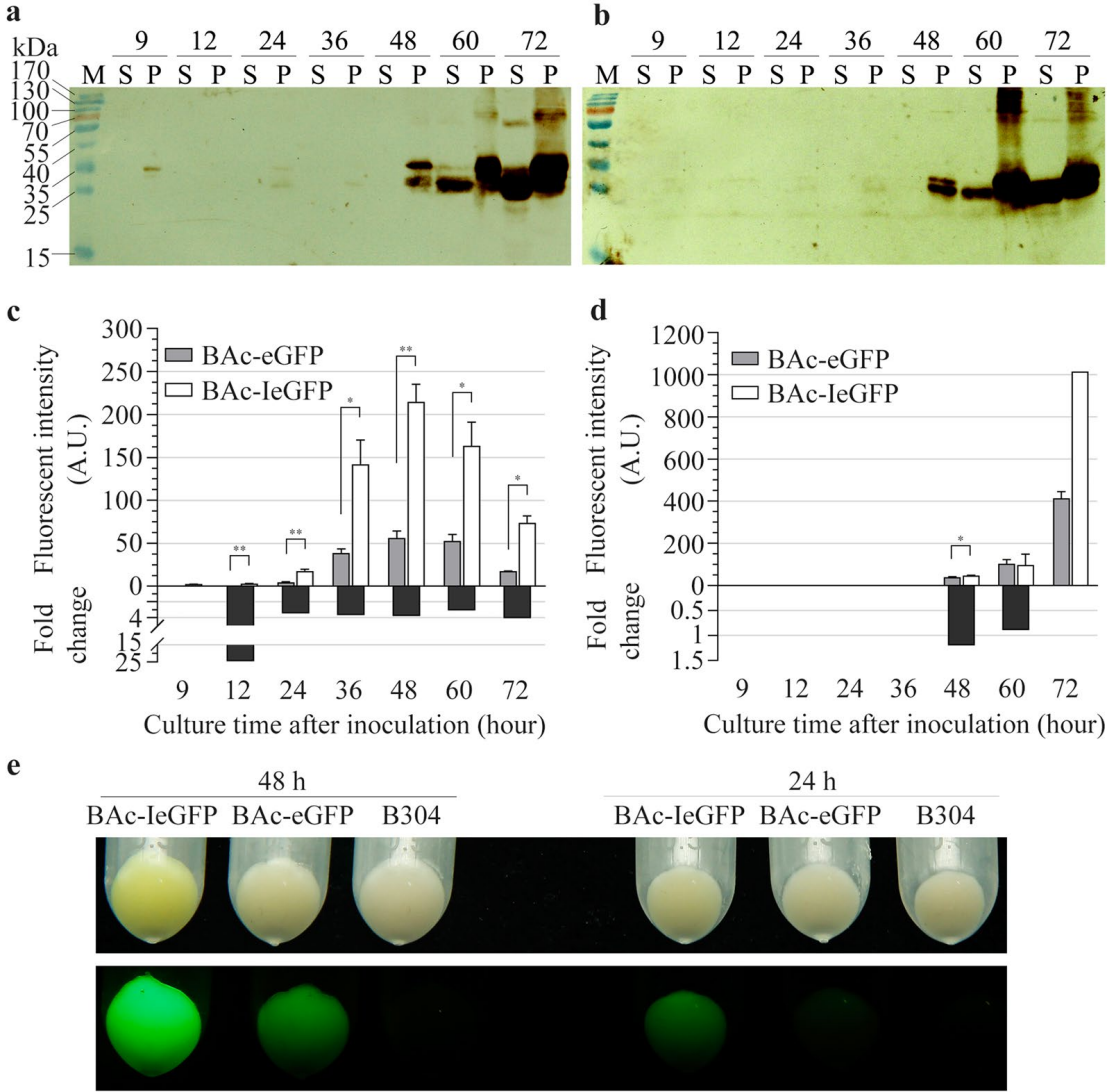
Expression analysis of eGFP and IeGFP regulated by the *P*_*ac*_ promoter in Bt strain. **a**, **b** The expression of IeGFP (panel **a**) and eGFP (panel **b**) in Bt at different times (9, 12, 24, 36, 48, 60 and 72 h after inoculation) were analyzed respectively by western blot. “S” represents the supernatant of cell culture and “P” represents the resuspended cells by PBS buffer. Lane “M” is the molecular weight standards. **c**, **d** The fluorescent intensities of the harvested cells (panel **c**) or the supernatants of cell cultures (panel **d**) and their fold changes for BAc-IeGFP over BAc-eGFP strain at corresponding culture time. The slit widths of EX/EM were both 3 nm and the detections were conducted in low sensitivity for the resuspended cells and in high sensitivity for the supernatant. The fluorescent signals of B304 cells cannot be detected and the fluorescent intensity of the 72 h supernatant of BAc-IeGFP strain cell culture was beyond the limit (1000 A.U.). The error bars indicate standard error of mean. The significant differences of the fluorescent intensities between BAc-IeGFP and BAc-eGFP strains at corresponding time were indicated by single asterisk (p < 0.05) or double asterisks (p < 0.01). **e** Comparison of the collected cells before (up) and after excitement (down) when they were incubated for 24 h and 48 h. The cells were excited by blue light using Luyor 3415RG hand held lamp

The expression and resulting fluorescent signals of IeGFP and eGFP in Bt were investigated. In Bt cells, the expression of IeGFP controlled by *P*_*ac*_ were detected at 9 h after inoculation in the collected cells (Fig. 3a and b). During 12 to 36 h, the degradation of IeGFP protein was observed. The main degradation product was an approximately 26 kDa peptide (Fig. 3a, b and Additional file 1: Figure S1A). From 48 to 72 h, the intact band of IeGFP protein accumulated continuously and almost half of them were cleaved into the 26 kDa band. The eGFP protein shown a similar expression pattern with IeGFP (Fig. 3b). The truncated peptide of eGFP was also about 26 kDa, the same as the degradation product of IeGFP in vivo. IeGFP protein could not be detected in the supernatant of cell culture of BAc-IeGFP strain until 60 h after inoculation. Surprisingly, eGFP protein also appeared in the supernatant of the BAc-eGFP strain at the same time. The main immune signal in the supernatant samples were also about 26 kDa which was very similar to the truncated IeGFP and eGFP inside the cell.

With increasing incubation time, the fluorescent intensity of the BAc-IeGFP and BAc-eGFP cell increased during the first 48 h, consistent with the immunoblot analysis (Fig. 3a–c and Additional file 1: Table S2). Thereafter, whilst the protein continued to accumulate, it was accompanied by a decline in fluorescent intensity. It was notable that the fluorescent intensities of BAc-IeGFP kept more than three times higher than that of BAc-eGFP throughout the detection period (Fig. 3c and Additional file 1: Table S2). Interestingly, the fluorescent signals of IeGFP or eGFP in the supernatant were detected at same time after 48 h of inoculation at high sensitivity (Fig. 3d and Additional file 1: Table S3). Similarly, the enhanced fluorescent intensity of the BAc-IeGFP strain was readily observable in the altered pigmentation of cell pellets (Fig. 3e).

The expression pattern of IeGFP and eGFP in *E. coli* TG1 and BL21-star (DE3) strain, and Bt BMB171 strain were compared by western blot analysis (Additional file 1: Figure S1A). The result revealed the stability of eGFP expressed either in *E. coli* TG1 or in BL21-star (DE3) strains. The pelB signal peptide can guide almost all of eGFP (pelB-eGFP) to the periplasmic space of *E. coli* and then the leader peptide was removed. However, most of IeGFP proteins remained intact and only a fraction of them were processed, especially when its expression was regulated by the *T7* promoter. In Bt strain, both eGFP and IeGFP protein were cut to a slightly lower molecular weight band (26 kDa) in cells or in supernatant of cell culture.

### Iasp enhanced the expression level of recombinant proteins

**Fig. 4:**
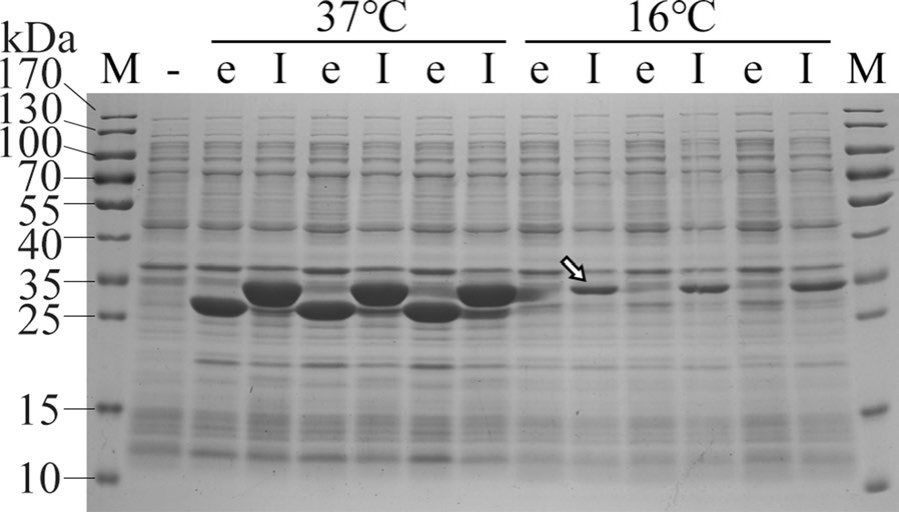
The expression analysis of IeGFP in *E. coli* BL21-star (DE3). IeGFP (lane “I”) and eGFP (lane “e”) expressed in *E. coli* BL21-star (DE3) at the different induction temperatures were analyzed by SDS-PAGE. Lane “-” is the negative controls sampled from the BL28aD cell culture and lane “M” is the molecular weight standards. The arrow indicates the IeGFP expressed at 16 °C

To confirm the effect of Iasp on improving the expression of recombinant proteins, the eGFP and IeGFP was expressed in *E. coli* BL21-star (DE3) strain, respectively. As a result, the *T7* promoter regulated the expression of IeGFP at higher level than eGFP at different temperatures, especially at 16 °C (Fig. 4). The expression level of eGFP was very low after 4 h induction at 16 °C, but the product can keep soluble (Additional file 1: Figure S2A). At the same condition, about half of the IeGFP products kept soluble. Nevertheless, the amount of this soluble fraction was higher than the total production of eGFP. It was notable that the expression level of IeGFP was similar to the NusA and MBP guided eGFP (N-eGFP and M-eGFP) and slightly higher than the Trx (thioredoxin) guided eGFP (T-eGFP). All of the fusion tags tested can produce considerable fraction of soluble fluorescent proteins in this study. For GDF8 and MMP13 proteins, their expressions were hard to identified by Coomassie brilliant blue stained SDS-PAGE analysis. However, with the assistance of Iasp or Trx, the expression levels were dramatically enhanced (I-MMP13, T-MMP13, I-GDF8 and T-GDF8) in *E. coli* BL21-star (DE3) strain (Fig. 4b and c). The difference was that almost all of I-MMP13, T-MMP13, I-GDF8 and T-GDF8 proteins were expressed as the inclusion bodies at the same induction condition (Additional file 1: Figure S2B and C).

The effect of the Iasp on its original protein Cry1Ia was also investigated. In Bt cells, the accumulation of the Cry1Ia (81.2 kDa) regulated by the *P*_*ac*_ promoter at 72 h after inoculation can be detected by SDS-PAGE (Additional file 1: Figure S3A). When removed the Iasp from the Cry1Ia, more accumulation of the truncated protein (Cry1IaD44, 76.2 kDa) was observed in Bt cells. Interestingly, Cry1IaD44 showed different degradation pattern compared to the intact Cry1Ia. The main degradation products of Cry1IaD44 (14 kDa) were soluble in the alkali buffer (50 mM Na_2_CO_3_, 10 mM dithiothreitol, pH 10.5) (Additional file 1: Figure S3B). Similar degraded fragment was observed in the expression product of a further truncated variant Cry1IaD73 (73.3 kDa), losing the N-terminal 73 amino acids of Cry1Ia. The result suggested the Iasp or its associated chaperone can prevent the attacking of specific protease(s). Interestingly, the different processing pattern observed in Bt cells was not detected in *E. coli* strain (Additional file 1: Figure S3C).

### Effect of Iasp encoding sequence on the transcript level of *Iegfp* gene

**Fig. 5:**
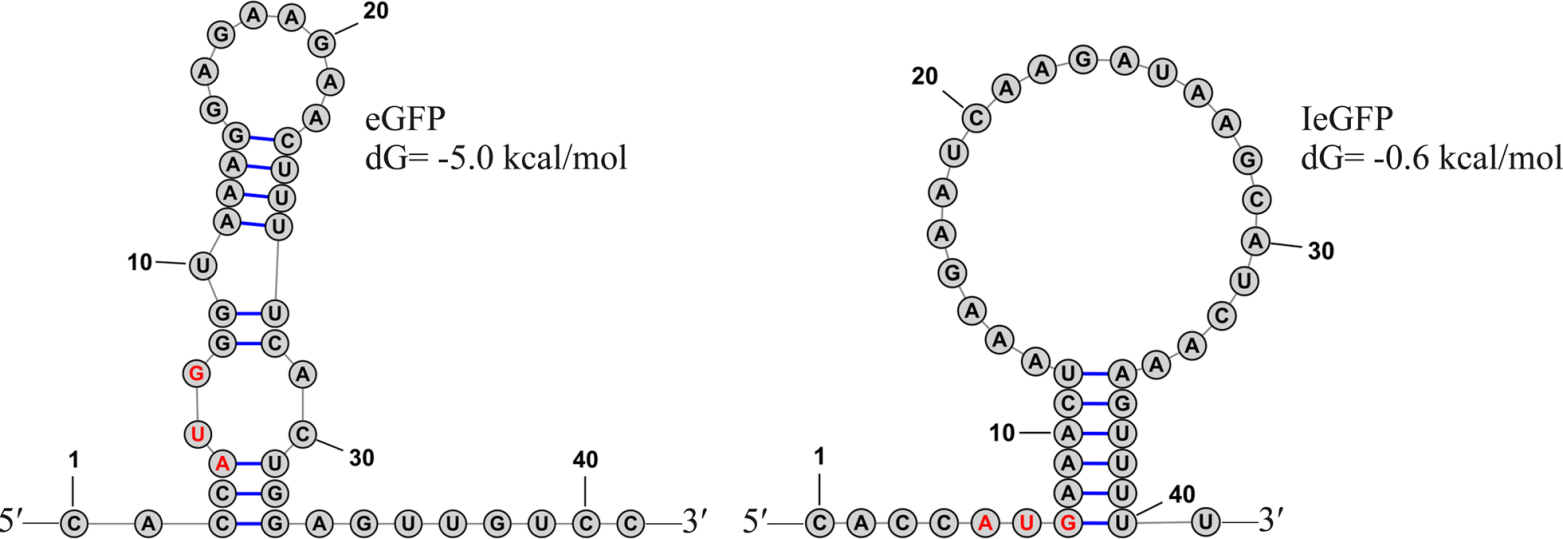
Prediction of the secondary structure of sequences near the initial codon. The secondary structure of sequences near the initial codon (−4 ~ + 37) of *egfp* (left) and *Iegfp* (right) gene were predicted by RNAstructure software. The initial codon is indicated in red. The folding energies (dG) are also indicated respectively

To identify the effect of IeGFP encoding sequence on the fusion gene, the transcription levels of the *Iegfp* and *egfp* genes in *E. coli* TAc-IeGFP and TAc-eGFP strains were compared using real-time quantitative PCR. As shown in Table 2, the transcription level of *Iegfp* gene in TAc-IeGFP strain was approximately 7 or 13 times higher than *egfp* gene in TAc-eGFP at 8 h or 12 h after inoculation. But interestingly, the wide difference in mRNA level between the *Iegfp* and *egfp* genes only resulted in the double protein level indicated by fluorescent intensity in Fig. 1. In addition, the structure of the 5′ leader sequence of the target mRNA plays an important role in affecting the translational efficiency, especially the nucleotides near the start codon (-4 to +37) [27]. We predicted the secondary structures of *Iegfp* and *egfp* genes by RNAstructure software [47]. The result showed that the calculated folding energy of the nucleotides near the start codon (− 4 to +37) of *Iegfp* was about − 0.6 kcal/mol, which implied less thermodynamic stability in this section than that of *egfp* gene (− 5 kcal/mol, Fig. 5).

**Table 2:** Comparison of *Iegfp* and *egfp* gene in mRNA level

No.	TAc-eGFP				TAc-IeGFP				Fold change
	Mean Ct of *hcat* gene	Mean Ct of *egfp* gene	Mean of 2^−ΔCt^	Variable coefficient (%)	Mean Ct of *hcat* gene	Mean Ct of *Iegfp* gene	Mean of 2^−ΔCt^	Variable coefficient (%)	
8 h									
1	26.91 ± 0.159	13.82 ± 0.131	989.466 ± 1018.333	10.289	30.18 ± 0.162	14.11 ± 0.118	75,603.881 ± 9462.533	12.516	7.639
2	27.12 ± 0.024	13.78 ± 0.200			30.42 ± 0.031	14.30 ± 0.153			
3	27.40 ± 0.076	14.03 ± 0.476			27.94 ± 0.033	11.54 ± 0.292			
12 h									
1	26.59 ± 0.136	14.73 ± 0.203	2945.768 ± 980.636	33.290	27.29 ± 0.088	11.86 ± 0.079	38,480.124 ± 14,483.546	37.639	13.639
2	27.21 ± 0.111	15.53 ± 0.353			26.68 ± 0.128	12.25 ± 0.120			
3	26.30 ± 0.058	15.46 ± 0.199			25.55 ± 0.086	9.97 ± 0.065			

The precision of the measures was indicated by the standard deviations

### Comparison the Iasp with the pelB and torA signal peptide

**Fig. 6:**
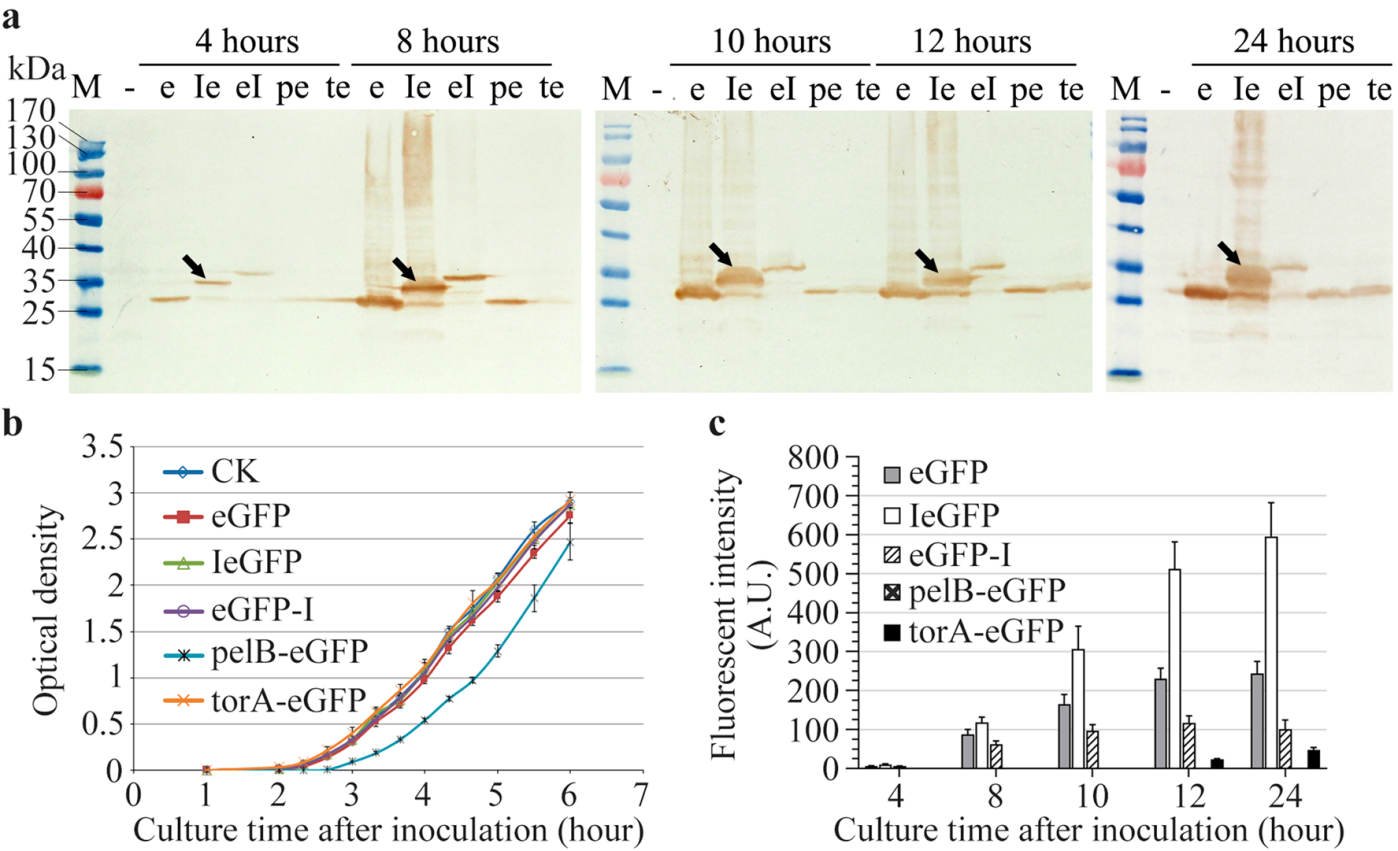
Expression analysis of the different fluorescent proteins in *E. coli* strain. **a** The expression of eGFP (lane “e”), IeGFP (lane “Ie”), eGFP-I (lane “eI”), pelB-eGFP (lane “pe”) and torA-eGFP (lane “te”) at different times (4, 8, 10, 12 and 24 h after inoculation) were analyzed by western blot. Lane “-” is the negative control which prepared from T304 cells sampled at the corresponding times respectively. Lane “M” is the molecular weight standards. The arrows indicate the expressed IeGFP at different times. **b** The optical densities at 600 nm (OD_600_) of the *E. coli* strains expressing the different fluorescent proteins were monitored during the first 6 h after inoculation. The error bars indicate standard deviation of mean. **c** The fluorescent intensities of the samples obtained at corresponding times in panel **a** (slit widths of EX/EM = 3/5 nm in low sensitivity). The fluorescent signals of T304 cells cannot be detected. The error bars indicate standard error of mean

The expression of IeGFP in *E. coli* was compared with the signal peptide of pelB (pectate lyase B) or torA guided eGFP (pelB-eGFP and torA-eGFP). The signal peptides of pelB or torA can guide the target protein into periplasm of *E. coli* by two representative inner membrane spanning transporters (SecYEG translocon or the twin-arginine translocation system) respectively [31]. Another fusion protein eGFP-I was also constructed to identify the function of Iasp when located at the C-terminal of eGFP. The expression of these proteins was monitored at five time points (4, 8, 10, 12 and 24 h after inoculation). The western blot analysis showed that all of these proteins can expressed under regulation of the constitutive promoter *P*_*ac*_ (Fig. 6a). The expression level of IeGFP after 10 h of incubation was higher than pelB-eGFP and torA-eGFP. But when the Iasp was located at C-terminal of eGFP (eGFP-I), the expression level decreased. Interestingly, the observed band of eGFP-I was larger than the expected molecular weight (33.1 kDa). Both of the signal peptides pelB and torA did not improve the expression level of eGFP but might translocate eGFP efficiently because the observed bands of pelB-eGFP and torA-eGFP were equal to the eGFP in molecular weight. The result indicated that both signal peptides were cleaved by corresponding peptidase after translocation. The growth and the fluorescent intensities of these strains were also monitored and the results were consistent with the immunoblot analysis except the strains expressing pelB-eGFP and torA-eGFP respectively (Fig. 6b, c and Additional file 1: Table S4). The expression of the pelB-eGFP negatively affected the growth of the *E. coli* cells and the expression products did not fluoresce normally. The expression level of torA-eGFP was lower than pelB-eGFP observed by western blot analysis, but its fluorescent signal was detected after 12 h of incubation.

### Subcellular location of IeGFP in *E. coli*

**Fig. 7:**
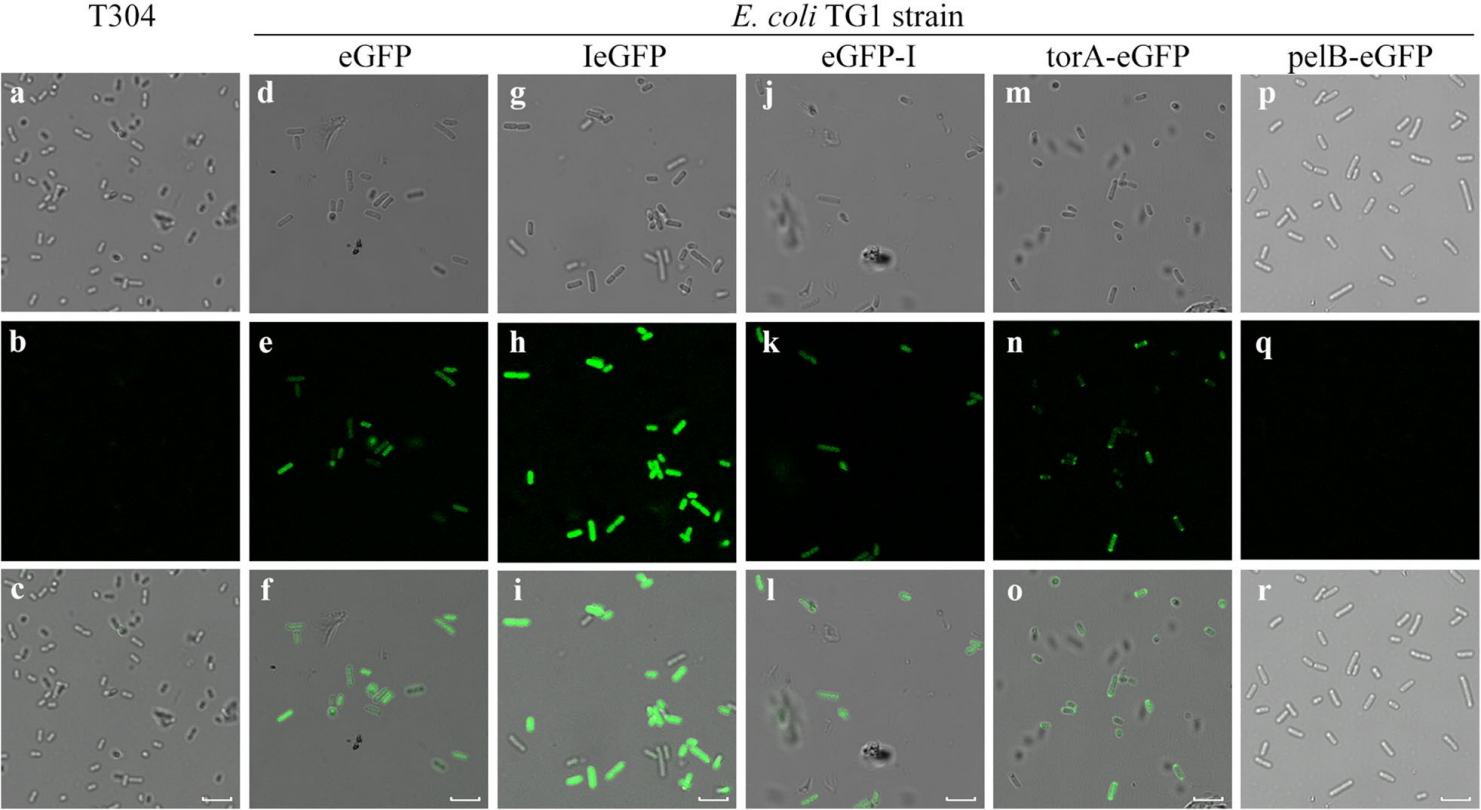
Confocal images analysis of *E. coli* cells expressing different fluorescent proteins. The *E. coli* cells expressing eGFP (**d**–**f**), IeGFP (**g**–**i**), eGFP-I (**j**–**l**), torA-eGFP (**m**–**o**) or pelB-eGFP (**p**–**r**) were observed by inverted confocal microscope (Leica SP8). The cells of T304 strain (**a**–**c**) were used as the negative control. The bright-field images and the fluorescent images were arranged in the first and second row, respectively, and then were merged (the third row). The scale bars represent 5 μm

According to the confocal images, the fluorescent signals were observed evenly in the cytoplasm for cells expressing eGFP, IeGFP and eGFP-I (Fig. 7). The fluorescent signal of pelB-eGFP, by contrast, was not detected in the host cells. For cells expressing torA-eGFP, the polar localization was observed.

### Verification of the *cry1Ia* gene promoter regulation using IeGFP

**Fig. 8:**
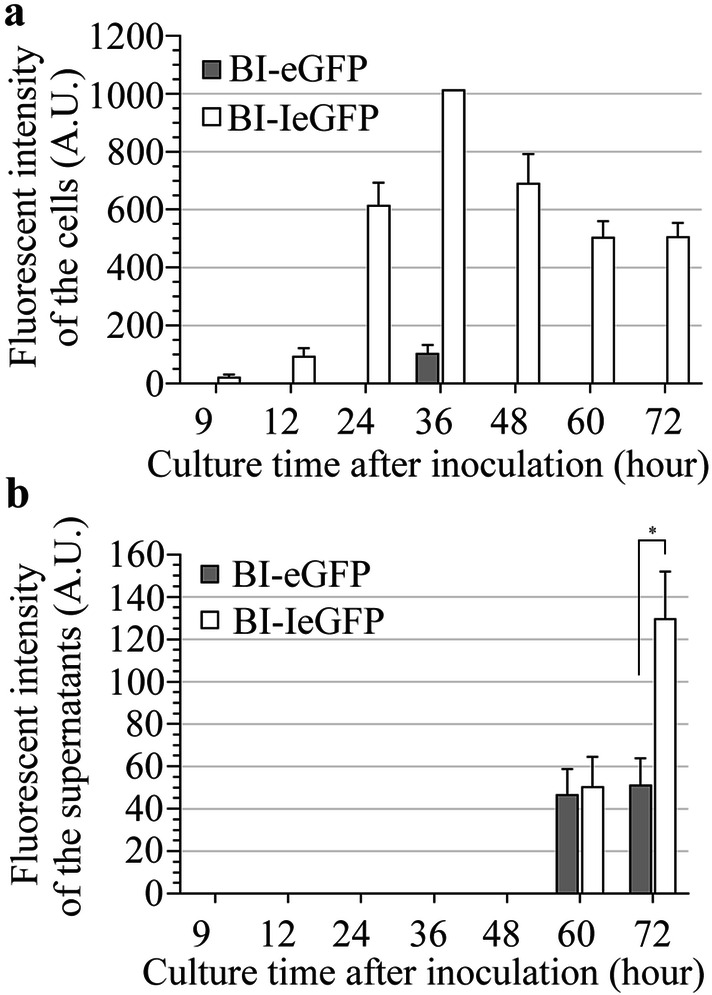
Fluorescent intensities analysis of the *P*_*i*_ promoter regulated eGFP and IeGFP in Bt strain. The fluorescent intensities of the resuspended cells by PBS buffer (**a**) and the supernatants of cell cultures (**b**) were detected respectively. The samples were obtained at different times (9, 12, 24, 36, 48, 60 and 72 h after inoculation). The slit widths of EX/EM were 3/3 nm and the detections were conducted in high sensitivity. The fluorescent signals of B304 cells cannot be detected and the fluorescent intensity of the 36 h cells of BI-IeGFP strain was beyond the limit (1000 A.U.). The significant difference of the fluorescent intensities between BI-IeGFP and BI-eGFP strains at corresponding time were indicated by single asterisk (p < 0.05). The error bars indicate standard error of mean

To confirm the better performance of IeGFP, the *Iegfp* and *gfp* genes were placed under control of the *cry1Ia* promoter (*P*_*i*_) and expressed in the Bt strain. The expression patterns of both proteins were identified and compared by the detection of fluorescent intensity. The result showed that the *P*_*i*_ promoter regulated the *Iegfp* gene expression in a similar way to that of *P*_*ac*_ over time (Fig. 8 and Additional file 1: Tables S5 and S6). But the expression level of *P*_*i*_ regulated genes was lower because the fluorescent signal of IeGFP and eGFP in BI-IeGFP and BI-eGFP cells was only detected on the 3/3 nm (the slit widths of EX/EM) in high sensitivity. According to the specification, the sensitivity of the spectrofluorophotometer (RF 5301PC) at high mode is about 50 times in comparison to that at low mode. In fact, the color of the cell pellets of BI-IeGFP and BI-eGFP were difficult to distinguish from the negative control cells by visible observation (B304 strain, data not shown).

### Verification of the function of Iasp on mCherry protein

**Fig. 9:**
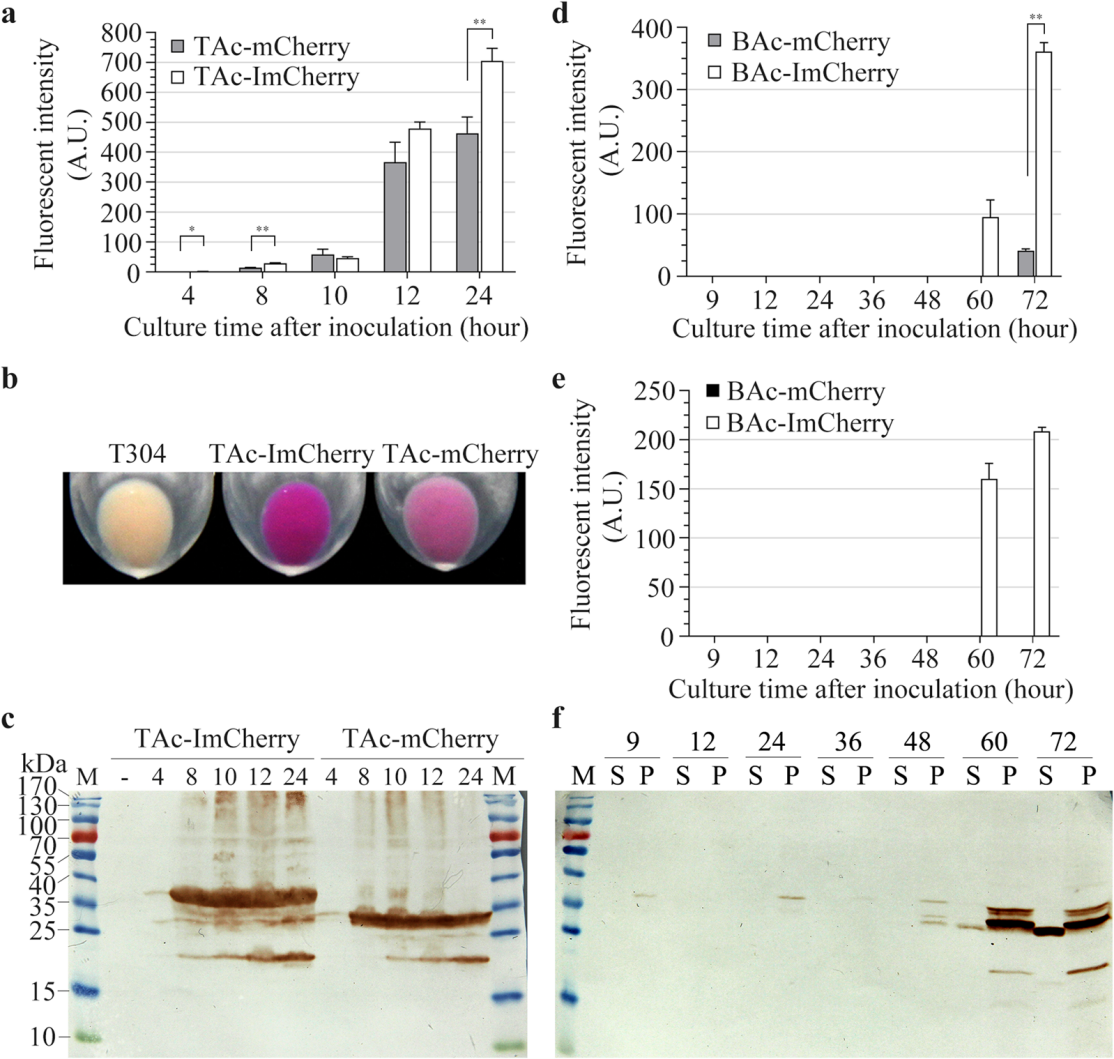
Expression analysis of mCherry and ImCherry in *E. coli* TG1 and Bt BMB171 strain. **a** The fluorescent intensities of the harvested TG1 cells expressing mCherry or ImCherry were monitored at different times (4, 8, 10, 12 and 24 h after inoculation). The slit widths of EX/EM were 1.5 nm and 3 nm, and the detections were conducted in high sensitivity. The fluorescent signals of B304 cells cannot be detected. The error bars indicate standard error of mean. The significant differences of the fluorescent intensities between TAc-ImCherry and TAc-mCherry cells at corresponding time were indicated by single asterisk (p < 0.05) or double asterisks (p < 0.01). **b** Comparison of the collected *E. coli* TG1 cells when they were incubated for 24 h. **c** Western blot analysis of the expression of mCherry and ImCherry in *E. coli* TG1 strain. “-” lane is negative control which prepared from T304 cells sampled at 12 h after inoculation. TAcImCherry and TAcmCherry cells were separately taken at 4, 8, 10, 12 and 24 h after inoculation. “M” represents the molecular weight standards. **d**, **e** The fluorescent intensities of the supernatants of cell cultures (panel D, slit widths of EX/EM = 3/5 nm in high sensitivity) or the harvested Bt cells (**e** slit widths of EX/EM = 3/3 nm in high sensitivity) at corresponding culture time. The fluorescent signals of B304 cells cannot be detected. The error bars indicate standard error of mean. The significant differences of the fluorescent intensities between BAc-ImCherry and BAc-mCherry cells at corresponding time were indicated by double asterisks (p < 0.01). **f** Western blot analysis of the expression of ImCherry in Bt BMB171 strain. “S” represents the supernatant of cell culture and the “P” means the resuspended cells by PBS buffer. “M” represents the molecular weight standards

To identify the effect on other FPs in vivo, the Iasp was used to guide the expression of mCherry. The new fusion protein ImCherry was also expressed in *E. coli* TG1 strain (TAc-ImCherry) and the Bt BMB171 strain (BAc-ImCherry), respectively, under control of *P*_*ac*_ promoter. Similar results to those with IeGFP were observed. The fluorescent signals of the TAc-ImCherry strain were more intensive than the corresponding control strain TAc-mCherry (Fig. 9a and Additional file 1: Table S7). The 24 h-after-inoculation cells of TAc-ImCherry strain were distinguished easily with the TAc-mCherry strain (Fig. 9b). Most of ImCherry proteins were expressed in the intact molecules (33.0 kDa) and only a fraction of them were digested into a 28 kDa band which was equal to the individually expressed mCherry protein (27.8 kDa, Fig. 9c).

In Bt cells, the fluorescent signal of mCherry cannot be detected during the cultivation period tested whereas the ImCherry performed well (Fig. 9c and e, and Additional file 1: Tables S8 and S9). The significant accumulation of ImCherry protein was also observed after 60 h of incubation, consistent with the fluorescent signal variation (Fig. 9f). The expression of the individual mCherry cannot be detected by western blot analysis even in 72 h-after-inoculation cells (data not shown). These results implied lower expression levels of the mCherry and ImCherry than the eGFP and IeGFP which might be attributed to the non-optimal codon used in the *mCherry* gene. Due to lower expression level in Bt strain, only weak violet color was observed for the BAc-ImCherry strain after 60 h inoculation (data not shown).

Interestingly, the immune signals of ImCherry were also detected in the supernatant of cell culture after 60 h of incubation and the corresponding bands were lower than the degraded products inside the cells (Fig. 9f and Additional file 1: Figure S1B). The band detected in the supernatant of cell culture of BAc-ImCherry strain was equal to the mCherry expressed in *E. coli* TG1 strain (Additional file 1: Figure S1B).

## Discussion

In this study, the predicted secretion signal peptide of Cry1Ia (Iasp) was fused to the N-terminal of eGFP and mCherry to produce novel fluorescent proteins, IeGFP and ImCherry. These fusion fluorescent proteins were not constructed for translocation across the membrane after expression, but to identify the positive effect of the Iasp on protein expression in the cytoplasm of prokaryotic cells.

Iasp can improve the expression level of eGFP and sequentially enhance the fluorescent signal of the tested prokaryotic cells. Under regulation of *P*_*ac*_ promoter, the fluorescent intensity of TAc-IeGFP strain was significantly higher than that of TAc-eGFP strain and so did the corresponding Bt strains. In *E. coli* cells tested, the expression of fluorescent proteins regulated by *P*_*ac*_ cannot be identified easily by SDS-PAGE analysis that suggested a weaker activity of *P*_*ac*_ than the *T7* promoter. But it was sufficient to alter the pigmentation of the host cells. Similarly, Iasp could also enhance the expression level of mCherry in the tested bacteria and the fluorescent intensity of the host cells. These results indicated that Iasp can improve the expression level of fluorescent proteins. The fusion fluorescent proteins, such as IeGFP and ImCherry constructed in this study, can accumulate inside host cells and therefore be used as novel fluorescent protein variants.

Iasp encoding sequence can affect the stability of mRNA and/or improve the translation efficiency. The observed high levels of transcripts of *Iegfp* gene were probably responsible for the enhanced expression level of IeGFP. In addition, Kudla et al. [27] reported that the structure of the 5′ leader sequence of the target mRNA played an important role in influencing the translational efficiency, especially the nucleotides near the initial codon (− 4 to + 37). The authors fused a 28-codon leader sequence with low secondary structure into the 5′ of different GFP encoding sequences. These fusion genes produced uniformly high expression levels. The strong correlation between mRNA folding energy and expression level suggests that the tightly folded messages obstruct translation initiation and thereby reduce protein synthesis [27, 48]. According to the prediction results of RNAstructure software [47], the participation of Iasp encoding sequence decreased the thermodynamic stability of the secondary structure near the initial codon (− 4 to + 37). Interestingly, in Kudla’s report, the *gfp* gene variants resulting high fluorescent intensities showed the similar folding energy level with the *egfp* construct used in this study (about − 5.0 kcal/mol). This implicated that Iasp encoding sequence could optimize the secondary structure of this region further and sequentially improve the translational efficiency. The result was also consistent with the report of Boël G et al. [49]. By high-throughput protein-expression analysis, the authors found that several factors, including the folding of mRNA head and the codon usage near this region, affected the translation efficiency in *E. coli*. The codon usage of 5′ coding sequence (CDS) does not only regulate the secondary structure of mRNA, but also influences the efficient ribosome docking. Therefore, Iasp can also be used as a fusion tag to improve the expression level of other recombinant proteins, such as two difficult-to-express proteins MMP13 and GDF8 tested in this study.

The interactions between the nascent peptide and the chaperones would alleviate the aggregation tendency of the highly expressed peptides and provide them more opportunities or directly assist them in folding into the correct 3D structure. For instance, co-expression of some chaperones and co-chaperones can prevent misfolding of eukaryotic proteins in prokaryotic system [50, 51]. Zhang et al. [25] reported that the combination of DnaK, DnaJ and GrpE (K/J/E) helped the folding of N-terminal nascent peptide and improved the solubility of the GFP partner in fusion protein. Consequently, the fluorescence of the fusion protein was enhanced in *E. coli*. Another chaperones, such as the novel Spy [26], or chaperone-interacted partner proteins, such as the classic MBP and NusA [17], can be used in the fusion proteins to enhance their solubility. For secretory proteins, the signal peptides also interact with corresponding chaperones which not only transfer the nascent peptides to the translocation machinery but also prevent aggregation. It was proven that alternating signal peptides or proper modification improved the expression-secretion efficiency of recombinant proteins simultaneously [52, 53]. However, some the signal peptides utilizing the SecYEG translocon, which only translocate the unfolded peptides, were reported to negatively affect the stability and folding of target proteins. For instance, the signal peptide of MBP (malE) and pelB led the thermodynamic destabilization of the C-terminal mature MBP or thioredoxin (Trx) due to the hydrophobic interactions between the signal peptides and the unfolded peptides. The interactions were thought necessary to hold the unfolded state before translocation [54–57]. Although the related secretory pathway of the Cry1Ia is unclear and the components participated in the machinery still keep uncovered, Iasp would interact with the corresponding chaperone(s) in its secretory pathway. In this study, soluble expression of IeGFP regulated by *T7* promoter was observed and the production yield of soluble fraction was higher than that of eGFP. The solubility enhancing effect of Iasp on eGFP was similar to the well-known fusion tags NusA, MBP and Trx. However, both Iasp or Trx fused MMP13 (I-MMP13 or T-MMP13) and GDF8 (I-GDF8 or T-GDF8) were expressed in insoluble form. The expression result of T-MMP13 and T-GDF8 was consistent with the previous report [19]. Therefore, the fitness of solubility enhancing effect of Iasp on diverse recombinant proteins need to be investigated further. Several hypotheses have been suggested to explain the mechanisms of solubility enhancement by fusion tags, including formation of micelle-like structures, attracting chaperons, exerting an intrinsic chaperon-like activity or net charged (reviewed in [17]). As a signal peptide, the interaction of Iasp with the corresponding chaperon(s) would be supposed and the deletion mutation on its original protein Cry1Ia might provide a clue. After removal of the Iasp, the truncated Cry1Ia proteins (Cry1IaD44) accumulated inside the Bt cells and showed different degradation profile compared to the intact Cry1Ia. Interestingly, in *E. coli* BL21-star (DE3) strain, no visible difference between them were observed by SDS-PAGE analysis besides the expected bands of Cry1Ia, Cry1Ia44 and Cry1Ia73. The result might be attribute to the different protease constitution between *E. coli* and Bt cells, and the phenomenon implied the effect of Iasp on preventing the Cry1Ia protein from attacking by some proteases directly or indirectly.

Iasp cannot translocate the eGFP efficiently in *E. coli*. By confocal microscopy, most of IeGFP proteins accumulated in the cytosol of *E. coli* cells that was consistent with the western blot analysis. According to the previous reports, the saturation of corresponding secretory pathway led to accumulation of recombinant proteins in cytosol [41, 42]. Although a fraction of IeGFP molecules were detected as a smaller band that probably represented the molecules translocated into periplasm, no fluorescent signal in this location were captured by confocal microscopy. Similarly, the expression of pelB-eGFP was detected by western blot analysis but no fluorescent signal was observed either in the cytoplasm or periplasmic space. Western blot analysis showed that almost all of expressed pelB-eGFP proteins were digested into a 27 kDa band that was equal to the individually expressed eGFP. Therefore, we concluded the efficiently translocation of eGFP guided by pelB signal peptide regulated by *P*_*ac*_. However, the high translocation efficiency of pelB-eGFP would overload the SecYEG-translocon and result in the growth retardation of TAc-pelBeGFP strain. Out of the known GFP variants, the sfGFP is the only one that can fold into its fluorescent form upon translocation via the SecYEG-translocon [15, 29, 30]. The post translocation folding mechanism was probably responsible for the observation for *E. coli* cells expressing pelB-eGFP. The Iasp would guide some eGFP peptide into periplasm via an analogous pathway besides that most of IeGFP peptides could not be captured by the corresponding chaperone(s) in their secretory pathway for unknown reasons, and then folded into the fluorescent form in the cytoplasm. The fluorescent signal of torA-eGFP can be detected albeit weak. The signal peptide of torA can translocate the target proteins into periplasm by Tat system. Unlike the SecYEG-translocon, the Tat system only guides the fully folded and/or co-factor incorporated proteins translocation [31, 32]. This different translocation mechanism explained the completely opposite fluorescing ability of pelB-eGFP and torA-eGFP in vivo. The polarization of fluorescence of torA-eGFP observed by confocal microscopy indicated the periplasm localization and the phenomenon resulted from the plasmolysis of *E. coli* cells in response to the osmotic up-shock when resuspended in PBS buffer [33]. In addition, when the Iasp was located at the C-terminal of eGFP (eGFP-I), its translocation function and effects on enhancing fluorescent signal were both lost.

In Bt cells, the IeGFP could not be translocated out of cells or in low efficiency because its immunoblot or fluorescent signal emerged at the same time point as the eGFP. The result indicated that the target proteins would not only be released by corresponding secretory pathway but also by the lysis of mother cells. Kostichka et al. [40] reported the Cry1Ia protein in natural host AB88 was not detected in pure crystal samples isolated from a 52 h culture but was detected in 26 h pellets of cells. Therefore, we speculated that some soluble Cry1Ia proteins stayed in the mother cells of Bt strain may release directly into supernatant during the spore maturation. The release of the soluble components would also be one of factors leading to the decline of fluorescent intensity of the resuspended precipitates of the BAc-eGFP and BAc-IeGFP cell cultures during last 12 h, especially at the 72 h after inoculation. The dramatically increased fluorescent intensity of 72 h supernatant of both BAc-eGFP and BAc-IeGFP could also support the speculation. Moreover, during the sporulation period, the *P*_*ac*_ promoter can accelerate the synthesis of the recombinant proteins that would prompt the formation of insoluble inclusion bodies. In this study, the rapid accumulations of eGFP and IeGFP after 48 h incubation were indicated by the immunoblot analysis. Therefore, the formation of inclusion bodies would be the second factor leading to the inconsistence between the significant decreasing of fluorescent intensity and the rapid accumulation of the protein products. The third factor would be the pH fluctuation in Bt cells at the latter sporulation stage. In *Bacillus subtilis*, the fluorescent intensity of a fusion proteins β-GFP, including the β subunit of prokaryotic RNA polymerase located at the N-terminal of GFP, was dramatically weaken during the sporulation stage V-VI by the decreased pH inside mother cells [58]. The period represented the last time before the mature spore release. The mCherry is more tolerant to a drop in pH [58]. Nevertheless, the fluorescent intensity of the resuspended BAc-IeGFP cells kept 3.5- to 4.1-fold times to that of BAc-eGFP during the whole culture period except the first 9 h, which were higher than the differences between the TAc-IeGFP and TAc-IeGFP (1.5- to 2.4-fold).

We also noticed the degradation of eGFP and IeGFP proteins in the Bt strains. In the cells at 9 or 12 h after incubation, the intact IeGFP or eGFP proteins were observed. But during the 12 to 36 h after inoculation, part of them were digested by unknown protease(s). After 48 h, the recombinant proteins accumulated rapidly and approximately half of them were processed by these protease(s). The genus *Bacillus* can produce diverse proteases and Bt is also an excellent source of proteases [59]. Generally, the overall protease activity of Bt strains follows a slow increase in the initial 12 h and then gradually increased to the peak at around 30 h after inoculation [59]. The protease activity variation would be partly responsible for the degradation process of the eGFP and IeGFP retained in Bt cells.

In supernatant of cell culture of corresponding Bt strains, the 26 kDa fragment, slightly smaller than the intact eGFP (27.9 kDa), was the main product. The N-terminal analysis revealed that the 26 kDa fragment started with GKGEELFT amino acids and therefore another cleavage site(s) would locate at the C-terminal of eGFP protein. The deficiency of the C-terminal of eGFP proteins would partially account for the weaker fluorescent intensity of eGFP and IeGFP in supernatant compared to the collected cells. Fox example, with loss of the C-terminal 11 amino acids, the expressed superfolder GFP 1-10 was insoluble and cannot fluoresce [60]. In addition, the immune signals of the ImCherry protein in the supernatant of cell culture and inside Bt cells were different in molecular weight. The result proved that there were different proteases responding to the degradation of ImCherry and IeGFP inside and outside the Bt cell. The intact ImCherry proteins expressed by the prokaryotic cells tested was slightly larger than its calculated molecular weight (33.0 kDa). This may be attributed to the unknown post-translational modification(s).

At present, little attention has been paid to the use of SPs with low translocation efficiency for recombinant protein production. The torA signal sequence was proven to direct expression of recombinant proteins in inclusion bodies even if the target was a well-soluble protein in *E. coli* [34]. This small inclusion body tag can help to express the toxic or unstable recombinant proteins. But the signal peptide of torA and Iasp would belong to different secretory pathways because the signal sequences of Tat system share the classic conversed sequence (S/T-RRXFLK), especially the double-arginine motif, which is not existed in Iasp [43–45]. More importantly, Iasp can guide the expression of eGFP at higher level than torA signal peptide under control of *P*_*ac*_ promoter in this study.

In conclusion, we identified the capacity of the SP of Cry1Ia protein (Iasp) in improving the expression of eGFP and mCherry proteins in *E. coli* and *Bt* cells. The insertion of encoding sequence of Iasp optimized the secondary structure near the initial codon and then facilitated the docking of ribosome. The more efficient recruitment of ribosome would account for the stability of mRNA and the rapid synthesis of the fusion fluorescent proteins. Meanwhile, Iasp can keep a considerable fraction of the fusion fluorescent proteins soluble inside the cells that guarantee the correct folding of fluorescent proteins. Therefore, the higher production of the soluble IeGFP and ImCherry generated the more intense fluorescent signal inside bacterial cells and these fusion proteins could be used as ideal candidates of fluorescent protein variants. Moreover, the function of Iasp on improving the expression of the recombinant proteins should also be highlighted and worthy of further investigation. This study also hinted the versatility of some signal peptides.

## Materials and methods

### Bacterial strains and growth conditions

All plasmids and strains used in this work are detailed in Table 1. Unless otherwise noted, *E. coli* cells were incubated in Luria–Bertani medium (LB medium) with 50 μg/mL ampicillin for pMD19 and pHT304 derived vectors, or 50 μg/mL kanamycin for pET28a derived vectors at 37 °C with 200 rpm shaking. Bt strains grew in LB medium (containing 25 μg/mL erythromycin if harboring the pHT304 plasmid or its derivatives) at 28.5 °C with 200 rpm shaking.

### Construction of expression vectors

*pET expression constructions* The *egfp* gene was amplified with primers EGFP-F and I/EGFP-R (Table 3) by PCR method and was TA-cloned into pMD19 vector (Takara, Tokyo, Japan). After sequence analysis, the BamHI/XhoI fragment of *egfp* gene was prepared and inserted into the corresponding site of the pET28aDel plasmid [61]. This vector is designated as p28aD-eGFP. The *Iegfp* gene was constructed by fusing Iasp encoding sequence (primers IEGFP-F and IEGFP-fuR) with *egfp* gene (primers IEGFP-fuF and I/EGFP-R) by overlapping PCR using primers IEGFP-F and I/EGFP-R (Table 3). The PCR product of *Iegfp* gene was cloned into pMD19 vector. After sequencing, the BamHI/XhoI restriction fragment of *Iegfp* gene was inserted into the corresponding site of the pET28aDel plasmid to form p28aD-IeGFP plasmid. The p22b-eGFP expression vector was constructed by ligating NcoI/SacI fragment of pET22b with same restriction fragment of *egfp* from the pAc-eGFP plasmid described below. The encoding sequences of matrix metalloprotease-13 (MMP13, genbank accession number AAP78940.1) and myostatin (growth differentiating factor-8, GDF8, genbank accession number 5F3B_C) genes and their fusion version I-MMP13, T-MMP13 (Trx-MMP13), T-GDF8 and I-GDF8 with Iasp (I) or Txr (T) were synthesized after codon optimization and then separately cloned into the BamHI/SacI site of the pET28aDel2 plasmid. The NcoI recognition site was added at the initial codon of the *mmp13* and *gdf8* genes. The pET28aDel2 was obtained by replacing the sequences between XbaI and BamHI of pET28a with 5′-AATAATTTTGTTTAACTTTAAGAAGGAGATATA. The T-eGFP encoding sequence was synthesized and inserted into the BamHI/SacI site the pET28aDel2 plasmid. The resulted plasmid was designated as p28aD2-T-eGFP. The p28aD2-M-eGFP and p28aD2-N-eGFP plasmids were constructed by replacing the *trx* sequence located between BamHI/NcoI site with the MBP and NusA encoding sequences, respectively. All of these expression vectors were separately transformed into BL21-star (DE3) strain by calcium chloride (CaCl_2_) transformation. Both the eGFP and IeGFP expressed by pET vectors described above were tagged by the His-tag at the C terminal.

**Table 3:** Primers used in this work

Primer	Sequence (5′-3′)
EGFP-F	ACGCGGATCCACCATGGGTAAAGGAGAAGAACTTTTCACTG
IEGFP-F	ACGCGGATCCACCATGAAACTAAAGAATCAAGAT
IEGFP-fuF	ATGAAGATTGTTTGAAAATGATGGGTAAAGGAGAAGAACT
IEGFP-fuR	AGTTCTTCTCCTTTACCCATCATTTTCAAACAATCTTCAT
I/EGFP-R	GACTCTCGAGTTTGTATAGTTCATCCATGCCA
304I/EGFP-R	GAGCTCTTAGTGGTGGTGGTGGTGGTGCT
Pac-F	GCCAAGCTTCAGGTAAATGGTTCTAACATGT
Pac-R	GGTGGATCCCCTCCATCTCTTTTATTAAGAT
Tac-F	GGATCCATCATCGAGCTCGAGGCAAACTCAGGTTTAAATATCGT
Tac-R	GGTACCACTGCACAATTGTATTGAATGAT
cry1Ia-F	ATAAAAGAGATGGAGGGATCCACCATGAAACTAAAGAATCAAGATAAG
cry1IaD44-F	ATAAAAGAGATGGAGGGATCCACCATGTCTGAGTATGAAAATGTAGAG
cry1IaD73-F	ATAAAAGAGATGGAGGGATCCACCATGCTAGGCGTTCCTTTTGCAGGACAAG
cry1Ia-R	AACCTGAGTTTGCCTCGAGCTCCTACATGTTACGCTCAATATGGAGT
Pi-F	CGCCAAGCTTGTATTTATAGGTGTTTGAAGT
Pi-R	TGGTGGATCCCCCTCCACTTATACTATTATTTAAT
pelBlaz-F	CTTAATAAAAGAGATGGAGGGGATCCACCATGAAATACCTGCTGCCGACC
pelBlaz-R	GAAAAGTTCTTCTCCTTTACCCATGGTGGATCTGGCCATCGCCGGCTGGGCAGCGAGGA
torAlaz-F	CTTAATAAAAGAGATGGAGGGGATCCACCATGAACAATAACGATCTCTTTCAG
torAlaz-R	GAAAAGTTCTTCTCCTTTACCCATGGTGGATCTCGCTTGCGCCGCAGTCGCACGTCG
egfpI-F	ATGGATGAACTATACAAACTCGAGCACCACCACCACCACCACATGAAACTAAAGAATCA
egfpI-R	TATTTAAACCTGAGTTTGCCTCGAGCTCTTACATTTTCAAACAATCTTCATGAT
mCherry-F	ACGCGGATCCACCATGGTGAGCAAGGGCGAGGAGGAT
ImC-fuF	ATCATGAAGATTGTTTGAAAATGATGGTGAGCAAGGGCGAG
ImC-fuR	CTCGCCCTTGCTCACCATCATTTTCAAACAATCTTCATGAT
304I/mC-R	TGGTGAGCTCTTAGTGGTGGTGGTGGTGGTGCTCGAGCTTGTACAGCTCGTCCATG
Hcat-F	CATCGGTCAACGGTACCA
Hcat-R	TGGCACTGCTGACACTTC
Hcat-Probe	FAM-AAGCCAATCATCACCAGCATCAGCCA-BHQ1
gfp-CF	GCCCTGTCCTTTTACCAGA
gfp-CR	CATCCATGCCATGTGTAATCC
gfp-Probe	FAM-CCATTACCTGTCCACACAATCTGCCCT-BHQ1

Restriction sites were underline

*pHT304 expression constructions* The pHT304 plasmid was linearized by SacI and blunted by T4 DNA polymerase (Thermo Fisher scientific, Grand Island, NY), and then recyclized by ligase to produce p304ΔSacI plasmid. Both the *egfp* (primers GFP-F and 304I/EGFP-R) and *Iegfp* (primers IEGFP-F and 304I/EGFP-R) gene were re-amplified by PCR method from the p28aD-eGFP and p28aD-IeGFP plasmids, respectively. Each PCR product contained the intact coding sequences (CDS) of the corresponding gene. The promoter (*P*_*ac*_, primers Pac-F and Pac-R) and terminator (*T*_*ac*_, primers Tac-F and Tac-R) of the *cry1Ac* gene were obtained by PCR method. The whole DNA extracted from a Bt strain preserved in the lab was used as the template. After sequencing, all of the *P*_*ac*_ fragment (HindIII/BamHI), the *T*_*ac*_ fragment (BamHI/KpnI) and the *egfp* gene or *Iegfp* gene (BamHI/SacI) were inserted one by one into the corresponding restriction sites of the p304ΔSacI plasmid. The plasmids were named pAc-eGFP or pAc-IeGFP. The *cry1Ia* gene (primers cry1Ia-F and cry1Ia-R) and its truncated variants *cry1IaD44* (primers cry1IaD44-F and cry1Ia-R) and *cry1IaD73* (primers cry1IaD73-F and cry1Ia-R) were amplified by PCR method using the Bt strain harboring a *cry1Ia* gene. These amplicons were cloned into the BamHI/SacI site of the pAc-eGFP, respectively, to obtain the pAc-Cry1Ia, pAc-Cry1IaD44 and pAc-Cry1IaD73 plasmids. The *cry1Ia*, *cry1Ia44* and *cry1Ia73* were also cloned into the BamHI/SacI site of pET28aDel plasmid for expression in the *E. coli* BL21-star (DE3) strain. The promoter of *cry1Ia* gene (*P*_*i*_) was obtained by PCR method using primers Pi-F and Pi-R and then was used to replace the *P*_*ac*_ sequence of pAc-eGFP or pAc-IeGFP with HindIII and BamHI. The resulting plasmids were designated as pI-eGFP and pI-IeGFP respectively.

The pAc-torAeGFP plasmid were constructed by inserting the *torA* fragment (the torA signal peptide encoding sequence) to the BamHI site of pAc-eGFP vector by ClonExpress II One Step Cloning Kit (Vazyme Biotech, Nanjing, China). The *torA* fragment was obtained by PCR method using torAlaz-F and torAlaz-R primers with the genomic DNA of *E. coli* TG1 strain as the template. The pAc-pelBeGFP plasmid was constructed by the same method except that the *pelB* fragment (the pelB signal peptide encoding sequence) was amplified by pelBlaz-F and pelBlaz-R primer with the pET22b plasmid as the template. Similarly, the amplicon of Iasp encoding sequence amplified by egfp-F and egfp-R primers was recombined into the XhoI site of pAc-eGFP vector to produce the pAc-eGFPI plasmid.

The plasmid pAc-mCherry was constructed by replacing the *egfp* gene in pAc-eGFP plasmid with *mcherry* fragment amplified using mCherry-F and 304I/mC-R primers. The template for the PCR reaction was pCMV-N-mCherry plasmid (Beyotime biotechnology, Beijing, China) which is used for protein expression in mammalian cells. The *ImCherry* gene was constructed by fusing Iasp encoding sequence (primers IEGFP-F and ImC-fuR) with *mCherry* gene (primers ImC-fuF and 304I/mC-R) by overlapping PCR using primers IEGFP-F and 304I/mC-R and then was inserted into the BamHI/SacI sites of the pAc-eGFP plasmid. The resulting plasmid was designated as pAc-ImCherry.

All of the pHT304, pAc-eGFP, pAc-IeGFP, pAc-Cry1Ia, pAc-Cry1IaD44 and pAc-Cry1IaD73, pI-eGFP, pI-IeGFP, pAc-pelBeGFP, pAc-torAeGFP, pAc-eGFPI, pAc-mCherry and pAc-ImCherry plasmids were transformed separately into calcium chloride (CaCl_2_) treated competent cells of *E. coli* TG1 strain and then into Bt BMB 171 strain by Bio-rad (Hercules, CA) MicroPulser™ Electroporator. The electroporation was performed using the 0.2 cm cuvette with 2.5 kilovolt pulse according to the manufacturer’s instructions.

### Proteins expression and samples preparation

*E. coli TG1 strain* The single colony of each *E. coli* TG1 strain harboring p304ΔSacI or its derived vectors described above was inoculated into 3 mL LB medium respectively and incubated overnight. The optical density at 600 nm (OD_600_) of cell cultures for all strains were adjusted to be equal and then 6 μL cell culture of each strain was collected and inoculated into 6 mL fresh LB medium. Unless otherwise noted, five repeats were inoculated for each strain. At 4, 8, 10, 12 or 24 h after inoculation, 1 mL of cell cultures from each repeat for each strain were taken and mixed. Out of the 5 mL mixture for each sample, 2 mL were separated and used to analyze the protein expression by western blot and the remaining 3 mL mixture were used for optical density and/or fluorescence detection. The analysis was replicated 3 times.

Bt *BMB 171 strain* The samples were prepared as same as the *E. coli* TG1 strains described above with the exception that each strains was sampled at 9, 12, 24, 36, 48, 60 or 72 h after inoculation. The cells and supernatant of the 5 mL mixture were separated by centrifugation. Then they were used for proteins expression analysis and fluorescence investigation. To express Cry1Ia and its truncated variants in Bt cells, the BAc-Cry1Ia, BAc-Cry1IaD44 and BAc-Cry1IaD73 strains were cultivated for 72 h and the total proteins in cells was analyzed by SDS-PAGE. In addition, the precipitates of BAc-Cry1Ia, BAc-Cry1IaD44 and BAc-Cry1IaD73 were resuspended by alkali buffer (50 mM Na_2_CO_3_, 10 mM dithiothreitol, pH 10.5). After 1 h incubation at 37 °C, the soluble and insoluble components were separated by centrifugation at 12,000 rpm and analyzed by SDS-PAGE.

*E. coli BL21*-*star (DE3) strain* The single colony of each BL21-star (DE3) strain was inoculated into 3 mL LB medium respectively and incubated at 37 °C with 250 rpm shaking overnight. Then 50 μL cell culture for each strain was pipetted into 50 mL fresh LB medium and continuously cultivated in the same condition. When the OD_600_ of the cell culture reached 0.8, the recombinant protein expression was induced by 1 mM IPTG (isopropyl β-D-1-thiogalactopyranoside) with 250 rpm shaking for 4 h. After induction, cells for each strain were harvested respectively by centrifuging at 10,000 rpm for 10 min and were washed by deionized water for three times. The cells were resuspended in 20 mL ice-cold LE buffer (50 mmol/L Na_2_HPO_4_, 0.3 mol/L NaCl, pH 8.0). Two milliliters of the cell suspension for each strain was pipetted into a new tube for protein analysis. The remaining cells were ultrasonic-treated in ice-cold condition. The lysates were centrifuged at 12,000 rpm at 4 °C for 15 min and then the supernatant and the resuspended precipitate by same volume of LE buffer as the supernatant of each strain was prepared for SDS-PAGE analysis.

### Fluorescence detection

The fluorescent signals were detected by Shimadzu RF530PC Spectrofluorophotometer (Kyoto, Japan). The excitation wavelength (EX) was 488 nm and the fluorescent intensities were recorded at 511 nm (emission wavelength, EM) for eGFP and IeGFP proteins. The EX/EM for detecting the mCherry were 565 nm and 603 nm, respectively. The fluorescent intensity is an arbitrary unit (A.U.). Before recording, the emission spectrum of each strain was scanned from 450 nm to 650 nm to confirm the emission peak was at or very close to the EM. The slit widths of EX and EM and the sensitivity of detection were indicated independently. The sensitivity at high mode is about 50 times higher than that at low mode according to the specification of RF 5301PC. For Bt strains, both the supernatant of the cell culture after centrifugation and the cells resuspended by fresh LB medium were monitored. For each *E. coli* strain, the whole cell culture was the loading sample. Each treatment for either *E. coli* or Bt strains was replicated three times. Unless otherwise noted, in each replication every record represented the value of mixture from 5 parallel tubes.

### Proteins analysis by SDS-PAGE and western blot

Unless otherwise noted, the collected cells of each sample from 2 mL culture were resuspended by 100 μL PBS buffer (137 mM NaCl, 2.7 mM KCl, 10 mM Na_2_HPO_4_, and 2 mM KH_2_PO_4_, pH 7.4) and the corresponding supernatant of Bt strains was concentrated to 200 μL by ultrafiltration using Amicon Ultra-0.5 Centrifugal Filters (3 kDa, Millipore, MA, USA). All of the protein samples were mixed with one-fourth volume of 5 × SDS gel-loading buffer respectively, and boiled for 5 min. After centrifuged at 12,000 rpm for 5 min, they were loaded onto gels for separation by SDS-PAGE and analyzed after Coomassie bright blue staining.

For western blot analysis, the separated proteins were transformed onto the nitrocellulose membrane without staining and incubated with rabbit antiserum against IeGFP protein and the horseradish peroxidase-conjugated goat antirabbit IgG (H + L) antibody (MultiSciences, HangZhou, China) successively. For detecting mCherry and ImCherry proteins, the primary antibody was the mCherry-tag monoclonal antibody (MultiSciences, HangZhou, China) and the secondary antibody was the horseradish peroxidase-conjugated goat anti-mouse IgG (H + L) antibody (MultiSciences, HangZhou, China). The target bands were visualized using 3,3′-diaminobenzidine (DAB, Sigma, St. Louis, MO).

### mRNA analysis of *Iegfp* and *egfp* gene

The transcription levels of *Iegfp* and *egfp* gene were compared by real-time quantitative PCR method. First, the total RNAs of TAc-eGFP and TAc-IeGFP cells collected at different times were extracted using RNAiso Plus (Takara Biomedical Technology, Beijing, China). Second, the obtained RNA samples were reverse-transcribed to cDNA by PrimeScript RT reagent kit with gDNA Eraser (Takara Biomedical Technology, Beijing, China). Finaly, the cDNAs were used for real-time PCR (Takara Premix Ex Taq for Probe qPCR) to quantify the transcription level of *egfp* and *Iegfp* genes. The *Hcat* gene of *E. coli* was used as the reference gene [62]. The primers used for real-time PCR were listed in Table 2. The differential expression analysis of *egfp* and *Iegfp* genes were evaluated by 2^−ΔCt^ method [63].

The secondary structures of nucleotides near the initial codon (−4 ~ + 37 nt) for both *Iegfp* and *egfp* genes were predicted by RNAstructure software [47]. The structure with lowest folding energy for each sequence were chosen for comparison.

### Fluorescence localization

The cells were prepared by Schlegel’s method with modification [15]. Briefly, cells corresponding to 1 A600 unit were harvested (4000×*g*, 2 min) and washed by 1 mL PBS buffer twice. Subsequently, 600 μL fixing solution (2% Paraformaldehyde, 2.5% Glutaraldehyde in PBS) was added and cells were incubated for 45 min at room temperature (RT). Subsequently, cells were washed three times with PBS and resuspended in 100 μL PBS. Three microliters of the cell suspension was mounted on a glass slide. Fluorescence images of cells were obtained using inverted confocal microscope (Leica SP8) with a 60 × times oil immersion objective (Leica). The resulting images were recorded with the LAS-AF-Lite software (Leica).

## Supplementary information

**Additional file 1: Table S1.** The fluorescent intensity data of TAc-eGFP and TAc-IeGFP cell cultures. **Table S2.** The fluorescent intensity data of the resuspended BAc-eGFP and BAc-IeGFP cells. **Table S3.** The fluorescent intensity data of the supernatant of BAc-eGFP and BAc-IeGFP cell cultures. **Table S4.** The fluorescent intensity data of five E. coli strains expressing eGFP and its variants. **Table S5.** The fluorescent intensity data of BI-eGFP and BI-IeGFP cells. **Table S6.** The fluorescent intensity data of the supernatants of BI-eGFP and BI-IeGFP cell cultures. **Table S7.** The fluorescent intensity data of TAc-mCherry and TAc-ImCherry cell cultures. **Table S8.** The fluorescent intensity data of supernatants of BAc-mCherry and BAc-ImCherry cell cultures. **Table S9.** The fluorescent intensity data of BAc-ImCherry cells. **Figure S1.** Comparison of fluorescent proteins expressed in different strains. **Figure S2.** Solubility analysis of three recombinant proteins. **Figure S3.** SDS-PAGE analysis of Cry1Ia and its truncated variants.

## Data Availability

All data generated or analyzed during this study are included in this published article.
