# Supplementary material for: The signal peptide of Cry1Ia can improve the expression of eGFP or mCherry in Escherichia coli and Bacillus thuringiensis and enhance the host’s fluorescent intensity
Source: Microb Cell Fact. 2020 May 24;19:112. doi: 10.1186/s12934-020-01371-8 (PMC7247199; doi:10.1186/s12934-020-01371-8)
Supplement: Supplementary file 1 — Additional file 1: Table S1. The fluorescent intensity data of TAc-eGFP and TAc-IeGFP cell cultures. Table S2. The fluorescent intensity data of the resuspended BAc-eGFP and BAc-IeGFP cells. Table S3. The fluorescent intensity data of the supernatant of BAc-eGFP and BAc-IeGFP cell cultures. Table S4. The fluorescent intensity data of five E. coli strains expressing eGFP and its variants. Table S5. The fluorescent intensity data of BI-eGFP and BI-IeGFP cells. Table S6. The fluorescent intensity data of the supernatants of BI-eGFP and BI-IeGFP cell cultures. Table S7. The fluorescent intensity data of TAc-mCherry and TAc-ImCherry cell cultures. Table S8. The fluorescent intensity data of supernatants of BAc-mCherry and BAc-ImCherry cell cultures. Table S9. The fluorescent intensity data of BAc-ImCherry cells. Figure S1. Comparison of fluorescent proteins expressed in different strains. Figure S2. Solubility analysis of three recombinant proteins. Figure S3. SDS-PAGE analysis of Cry1Ia and its truncated variants. [file 12934_2020_1371_MOESM1_ESM.docx]

**The signal peptide of Cry1Ia can improve the expression level of eGFP or mCherry in *Escherichia coli* and *Bacillus thuringiensis* and enhance the host’s fluorescent intensity**

Jianhua Gao^1^#, Hongmei Qian^1^, Xiaoqin Guo^1^, Yi Mi^1^, Junpei Guo^1^, Juanli Zhao^1^, Chao Xu^2^, Ting Zheng^2^, Ming Duan^3^, Zhongwei Tang^1^, Chaoyang Lin^2^, Zhicheng Shen^2^, Yiwei Jiang^4^, Xingchun Wang^1^#

^1^College of Life Sciences, Shanxi Agricultural University, Taigu, 030801, China. ^2^State Key Laboratory of Rice Biology, Institute of Insect Sciences, College of Agriculture and Biotechnology, Zhejiang University, Hangzhou, 310058, China. ^3^Experimental Teaching Center, Shanxi Agricultural University, Taigu, 030801, China. ^4^Department of Agronomy, Purdue University, United States.

#Address correspondence to Jianhua Gao: gaojh_edu@163.com or jhgao@sxau.edu.cn; Xingchun Wang: wxingchun@163.com or wxingchun@sxau.edu.cn

Table S1 The fluorescent intensity data of TAc-eGFP and TAc-IeGFP cell cultures

| Strains | Culture Time （Hour） | Fluorescent intensity (A.U.) | | | Mean | SEM | Fold change^a^ |
| --- | --- | --- | --- | --- | --- | --- | --- |
|  |  | 1 | 2 | 3 |  |  |  |
| TAc-eGFP | 4 | 3.150 | ND^b^ | 5.460 | 2.870 | 1.582 |  |
|  | 8 | 74.191 | 58.403 | 88.125 | 73.573 | 8.586 |  |
|  | 10 | 126.786 | 131.080 | 159.968 | 139.278 | 10.419 |  |
|  | 12 | 162.001 | 187.411 | 329.123 | 226.178 | 51.992 |  |
|  | 24 | 307.213 | 164.295 | 326.898 | 266.135 | 51.236 |  |
| TAc-IeGFP | 4 | 5.412 | ND | 7.564 | 4.325 | 2.250 | 1.5 |
|  | 8 | 103.742 | 100.950 | 119.294 | 107.995 | 5.707 | 1.5 |
|  | 10 | 284.536 | 352.418 | 368.954 | 335.303 | 25.828 | 2.4 |
|  | 12 | 342.677 | 598.022 | 516.916 | 485.872 | 75.328 | 2.1 |
|  | 24 | 448.777 | 610.444 | 504.919 | 521.380 | 47.389 | 2.0 |

a. The fold change for the fluorescence intensity of TAc-IeGFP over TAc-eGFP at corresponding time.

b. ND, not detected.

Table S2 The fluorescent intensity data of the resuspended BAc-eGFP and BAc-IeGFP cells

| Strains | Culture Time （Hour） | Fluorescent intensity (A.U.) | | | Mean | SEM | Fold Change^a^ |
| --- | --- | --- | --- | --- | --- | --- | --- |
|  |  | 1 | 2 | 3 |  |  |  |
| BAc-eGFP | 9 | ND^b^ | ND | ND | N/A^c^ | N/A |  |
|  | 12 | 0.119 | 0.128 | 0.190 | 0.146 | 0.022 |  |
|  | 24 | 4.164 | 5.657 | 5.814 | 5.212 | 0.526 |  |
|  | 36 | 40.288 | 46.564 | 29.557 | 38.803 | 4.965 |  |
|  | 48 | 40.460 | 63.440 | 65.972 | 56.624 | 8.115 |  |
|  | 60 | 42.566 | 49.147 | 67.774 | 53.162 | 7.549 |  |
|  | 72 | 17.386 | 17.862 | 18.422 | 17.890 | 0.299 |  |
| BAc-IeGFP | 9 | 3.072 | 2.296 | 2.904 | 2.757 | 0.236 |  |
|  | 12 | 3.417 | 3.770 | 3.599 | 3.595 | 0.102 | 24.7 |
|  | 24 | 22.273 | 15.683 | 16.340 | 18.099 | 2.096 | 3.5 |
|  | 36 | 182.031 | 88.008 | 156.761 | 142.267 | 28.093 | 3.7 |
|  | 48 | 249.708 | 215.756 | 178.216 | 214.560 | 20.647 | 3.8 |
|  | 60 | 108.692 | 190.453 | 191.928 | 163.691 | 27.503 | 3.1 |
|  | 72 | 58.067 | 83.970 | 80.048 | 74.028 | 8.061 | 4.1 |

a. The fold change for the fluorescence intensity of BAc-IeGFP over BAc-eGFP at corresponding time.

b. ND, not detected.

c. N/A, not available.

Table S3 The fluorescent intensity data of the supernatant of BAc-eGFP and BAc-IeGFP cell cultures

| Strains | Culture Time （Hour） | Fluorescent intensity (A.U.) | | | Mean | SEM | Fold Change^a^ |
| --- | --- | --- | --- | --- | --- | --- | --- |
|  |  | 1 | 2 | 3 |  |  |  |
| BAc-eGFP | 9 | ND^b^ | ND | ND | N/A^c^ | N/A |  |
|  | 12 | ND | ND | ND | N/A | N/A |  |
|  | 24 | ND | ND | ND | N/A | N/A |  |
|  | 36 | ND | ND | ND | N/A | N/A |  |
|  | 48 | 36.188 | 42.712 | 41.987 | 40.296 | 2.064 |  |
|  | 60 | 140.909 | 74.189 | 95.434 | 103.511 | 19.679 |  |
|  | 72 | 432.932 | 354.789 | 456.994 | 414.905 | 30.850 |  |
| BAc-IeGFP | 9 | ND | ND | ND | N/A | N/A |  |
|  | 12 | ND | ND | ND | N/A | N/A |  |
|  | 24 | ND | ND | ND | N/A | N/A |  |
|  | 36 | ND | ND | ND | N/A | N/A |  |
|  | 48 | 50.606 | 49.227 | 45.344 | 48.392 | 1.575 | 1.2 |
|  | 60 | 200.755 | 37.05 | 55.816 | 97.874 | 51.725 | 0.9 |
|  | 72^d^ | 1015.000 | 943.421 | 1015.000 | 991.140 | 23.860 | 2.4 |

a. The fold change for the fluorescence intensity of BAc-IeGFP over BAc-eGFP at corresponding time.

b. ND, not detected.

c. N/A, not available.

d. The fluorescent intensity of the 72 hours supernatant of BAc-IeGFP strain was beyond the limit (1000 A.U.). The average of fluorescent intensity of the supernatant of BAc-IeGFP would be more 2.4-fold than that of BAc-eGFP.

Table S4 The fluorescent intensity data of five *E. coli* strains expressing eGFP and its variants^a^

| Strains | Culture Time （Hour） | Fluorescent intensity (A.U.) | | | Mean | SEM |
| --- | --- | --- | --- | --- | --- | --- |
|  |  | 1 | 2 | 3 |  |  |
| TAc-eGFP | 4 | 5.700 | 7.830 | 7.745 | 7.092 | 1.206 |
|  | 8 | 63.609 | 104.062 | 98.317 | 88.663 | 21.886 |
|  | 10 | 122.194 | 207.317 | 166.938 | 165.483 | 42.580 |
|  | 12 | 188.365 | 281.152 | 221.293 | 230.270 | 47.040 |
|  | 24 | 199.277 | 303.719 | 229.446 | 244.147 | 53.751 |
| TAc-IeGFP | 4 | 10.211 | 13.563 | 8.807 | 10.860 | 2.444 |
|  | 8 | 104.254 | 147.121 | 102.821 | 118.065 | 25.173 |
|  | 10 | 222.049 | 419.466 | 278.902 | 306.806 | 101.623 |
|  | 12 | 384.172 | 622.062 | 530.511 | 512.248 | 119.992 |
|  | 24 | 427.941 | 718.224 | 640.131 | 595.432 | 150.215 |
| TAc-eGFPI | 4 | 7.145 | 7.013 | 6.001 | 6.720 | 0.626 |
|  | 8 | 45.794 | 70.203 | 71.912 | 62.636 | 14.611 |
|  | 10 | 68.083 | 102.913 | 121.551 | 97.516 | 27.140 |
|  | 12 | 83.446 | 124.723 | 144.538 | 117.569 | 31.168 |
|  | 24 | 58.532 | 109.027 | 138.249 | 101.936 | 40.329 |
| TAc-torAeGFP | 4 | ND^b^ | ND | ND | N/A^c^ | N/A |
|  | 8 | ND | ND | ND | N/A | N/A |
|  | 10 | ND | ND | ND | N/A | N/A |
|  | 12 | 27.281 | 19.346 | 23.801 | 23.476 | 3.977 |
|  | 24 | 54.517 | 37.345 | 53.990 | 48.617 | 9.766 |

a. The fluorescent intensity data of TAc-pelBeGFP cells cannot be detected at EX/EM=3/5 nm in low sensitivity during the cultivation period.

b. ND, not detected.

c. N/A, not available.

Table S5 The fluorescent intensity data of BI-eGFP and BI-IeGFP cells

| Strains | Culture Time （Hour） | Fluorescent intensity (A.U.) | | | Mean | SEM |
| --- | --- | --- | --- | --- | --- | --- |
|  |  | 1 | 2 | 3 |  |  |
| BI-eGFP | 9 | ND^a^ | ND | ND | N/A^b^ | N/A |
|  | 12 | ND | ND | ND | N/A | N/A |
|  | 24 | ND | ND | ND | N/A | N/A |
|  | 36 | 60.959 | 94.388 | 159.665 | 105.004 | 28.984 |
|  | 48 | ND | ND | ND | N/A | N/A |
|  | 60 | ND | ND | ND | N/A | N/A |
|  | 72 | ND | ND | ND | N/A | N/A |
| BI-IeGFP | 9 | 35.706 | 24.974 | 9.544 | 23.408 | 7.593 |
|  | 12 | 135.547 | 105.455 | 49.549 | 96.850 | 25.196 |
|  | 24 | 753.793 | 608.640 | 483.324 | 615.252 | 78.148 |
|  | 36^c^ | 1015.000 | 1015.000 | 1015.000 | N/A | N/A |
|  | 48 | 881.187 | 655.331 | 541.875 | 692.798 | 99.726 |
|  | 60 | 597.552 | 511.224 | 406.909 | 505.228 | 55.115 |
|  | 72 | 588.201 | 508.224 | 427.623 | 508.016 | 46.355 |

a. ND, not detected.

b. N/A, not available.

c. The fluorescent intensity of the 36 hours cells of BI-IeGFP strain was beyond the limit (1000 A.U.).

Table S6 The fluorescent intensity data of the supernatants of BI-eGFP and BI-IeGFP cell cultures

| Strains | Culture Time （Hour） | Fluorescent intensity (A.U.) | | | Mean | SEM | Fold Change^a^ |
| --- | --- | --- | --- | --- | --- | --- | --- |
|  |  | 1 | 2 | 3 |  |  |  |
| BI-GFP | 9 | ND^b^ | ND | ND | N/A^c^ | N/A |  |
|  | 12 | ND | ND | ND | N/A | N/A |  |
|  | 24 | ND | ND | ND | N/A | N/A |  |
|  | 36 | ND | ND | ND | N/A | N/A |  |
|  | 48 | ND | ND | ND | N/A | N/A |  |
|  | 60 | 25.858 | 47.339 | 67.383 | 46.860 | 11.990 |  |
|  | 72 | 30.175 | 50.983 | 73.336 | 51.498 | 12.462 |  |
| BI-IGFP | 9 | ND | ND | ND | N/A | N/A |  |
|  | 12 | ND | ND | ND | N/A | N/A |  |
|  | 24 | ND | ND | ND | N/A | N/A |  |
|  | 36 | ND | ND | ND | N/A | N/A |  |
|  | 48 | ND | ND | ND | N/A | N/A |  |
|  | 60 | 33.552 | 40.306 | 78.164 | 50.674 | 13.883 | 1.1 |
|  | 72 | 90.776 | 132.056 | 167.084 | 129.972 | 22.053 | 2.5 |

a. The fold change for the fluorescence intensity of BI-IeGFP over BI-eGFP at corresponding time.

b. ND, not detected.

c. N/A, not available.

Table S7 The fluorescent intensity data of TAc-mCherry and TAc-ImCherry cell cultures

| Strains | Culture Time （Hour） | Fluorescent intensity (A.U.) | | | Mean | SEM | Fold change^a^ |
| --- | --- | --- | --- | --- | --- | --- | --- |
|  |  | 1 | 2 | 3 |  |  |  |
| TAc-mCherry | 4 | 1.338 | 1.465 | 2.073 | 1.625 | 0.227 |  |
|  | 8 | 13.110 | 14.738 | 16.221 | 14.690 | 0.898 |  |
|  | 10 | 25.422 | 81.641 | 70.331 | 59.131 | 17.168 |  |
|  | 12 | 274.119 | 326.361 | 497.930 | 366.137 | 67.600 |  |
|  | 24 | 367.909 | 469.131 | 554.171 | 463.737 | 53.837 |  |
| TAc-ImCherry | 4 | 1.708 | 2.278 | 2.728 | 2.238 | 0.295 | 1.4 |
|  | 8 | 24.712 | 29.673 | 32.307 | 28.897 | 2.227 | 2.0 |
|  | 10 | 54.185 | 39.541 | 45.651 | 46.459 | 4.247 | 0.8 |
|  | 12 | 459.275 | 522.614 | 457.626 | 479.838 | 21.393 | 1.3 |
|  | 24 | 634.176 | 701.464 | 778.976 | 704.872 | 41.835 | 1.5 |

a. The fold change for the fluorescence intensity of TAc-ImCherry over TAc-mCherry at corresponding time.

Table S8 The fluorescent intensity data of supernatants of BAc-mCherry and BAc-ImCherry cell cultures

| Strains | Culture Time （Hour） | Fluorescent intensity (A.U.) | | | Mean | SEM | Fold Change^a^ |
| --- | --- | --- | --- | --- | --- | --- | --- |
|  |  | 1 | 2 | 3 |  |  |  |
| BAc-mCherry | 9 | ND^b^ | ND | ND | N/A^c^ | N/A |  |
|  | 12 | ND | ND | ND | N/A | N/A |  |
|  | 24 | ND | ND | ND | N/A | N/A |  |
|  | 36 | ND | ND | ND | N/A | N/A |  |
|  | 48 | ND | ND | ND | N/A | N/A |  |
|  | 60 | ND | ND | ND | N/A | N/A |  |
|  | 72 | 40.918 | 37.115 | 46.351 | 41.461 | 2.680 |  |
| BAc-ImCherry | 9 | ND | ND | ND | N/A | N/A |  |
|  | 12 | ND | ND | ND | N/A | N/A |  |
|  | 24 | ND | ND | ND | N/A | N/A |  |
|  | 36 | ND | ND | ND | N/A | N/A |  |
|  | 48 | ND | ND | ND | N/A | N/A |  |
|  | 60 | 65.175 | 150.288 | 69.718 | 95.060 | 27.645 |  |
|  | 72 | 384.175 | 364.824 | 333.100 | 360.700 | 14.888 | 8.7 |

a. The fold change for the fluorescence intensity of BAc-ImCherry over BAc-mCherry at corresponding time.

b. ND, not detected.

c. N/A, not available.

Table S9 The fluorescent intensity data of BAc-ImCherry cells^a^

| Strains | Culture Time （Hour） | Fluorescent intensity (A.U.) | | | Mean | SEM |
| --- | --- | --- | --- | --- | --- | --- |
|  |  | 1 | 2 | 3 |  |  |
| BAc-ImCherry | 9 | ND^b^ | ND | ND | N/A^c^ | N/A |
|  | 12 | ND | ND | ND | N/A | N/A |
|  | 24 | ND | ND | ND | N/A | N/A |
|  | 36 | ND | ND | ND | N/A | N/A |
|  | 48 | ND | ND | ND | N/A | N/A |
|  | 60 | 182.083 | 129.42 | 169.071 | 160.191 | 15.838 |
|  | 72 | 212.338 | 212.734 | 200.352 | 208.475 | 4.063 |

a. The fluorescent intensity data of BAc-mCherry cells cannot be detected at EX/EM=3/3 nm in high sensitivity during the cultivation period.

b. ND, not detected.

c. N/A, not available.


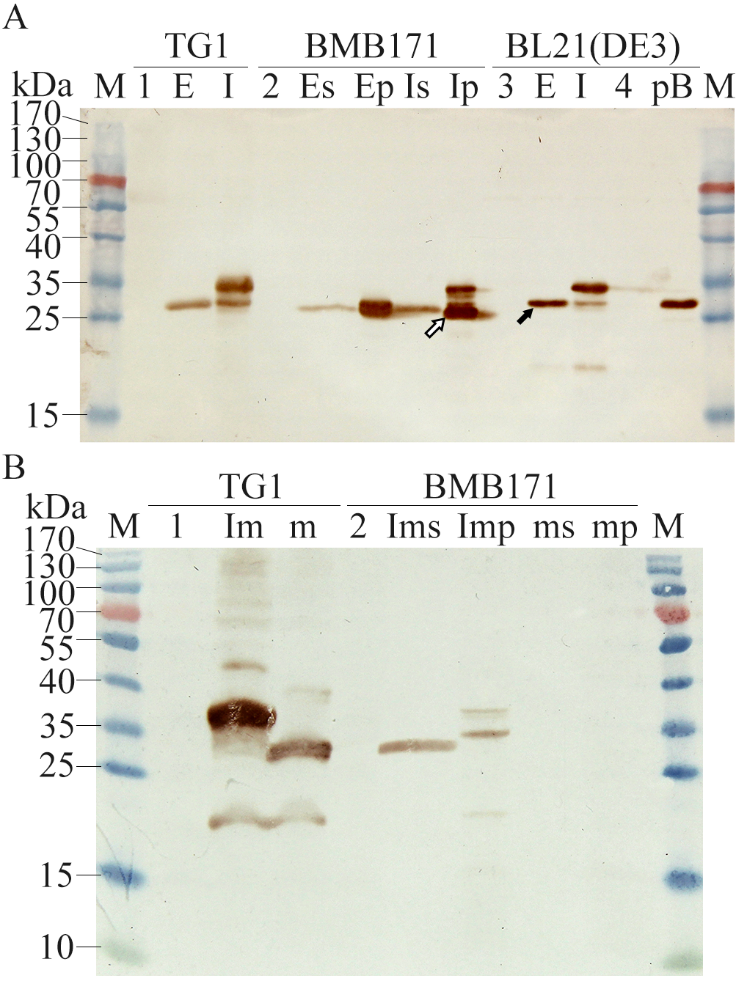


**Figure S1 Comparison of fluorescent proteins expressed in different strains**

**A:** Western blot analysis of IeGFP and eGFP expressed by *E. coli* TG1 and BL21-star (DE3), and Bt BMB171 strains. Lanes “1-4” are the negative controls for the corresponding expression strains sampled from the T304、B304、BL28aD and BL22b cell culture, respectively. “E” represents eGFP and “I” means IeGFP. “s” and “p” for Bt BMB171 strains represent separately the supernatant and precipitation of cell culture of Bt strains. “pB” means pelB-eGFP expressed by pET22b vector. Lane “M” is the molecular weight standards. The black arrow indicates the intact eGFP protein and the hollow arrow points out the 26 kDa product of processed IeGFP protein in Bt strain.

**B:** Comparison of expression pattern of ImCherry and mCherry in *E. coli* TG1 strain and Bt BMB171 strain. “1” and “2” lanes are negative controls for the corresponding expression strains sampled from the T304 and B304 cell culture, respectively. “m” represents mCherry and “Im” means ImCherry. “s” and “p” represent separately the supernatant and precipitation of cell culture of Bt strains. “M” represents the molecular weight standards.


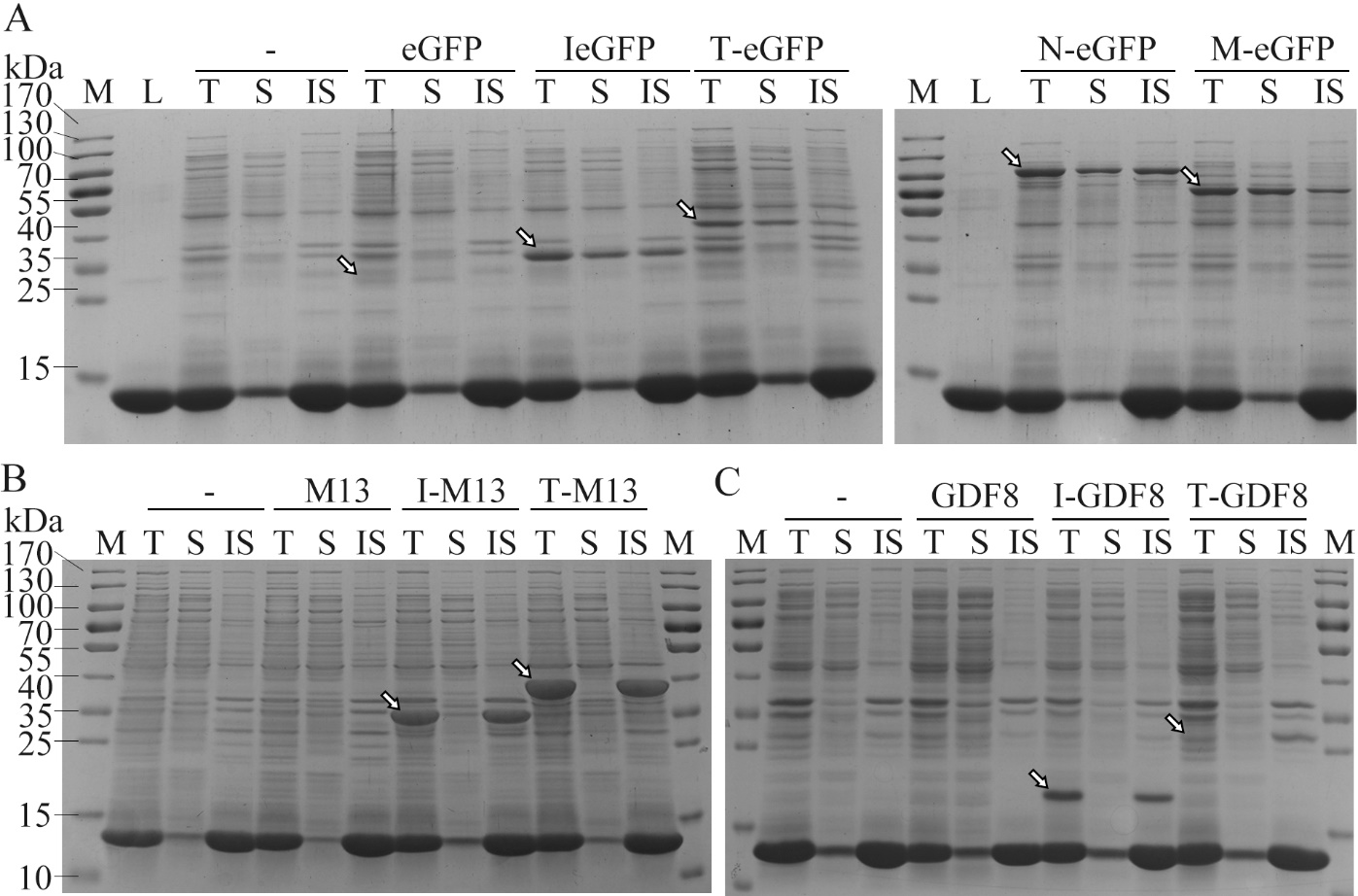


**Figure S2 Solubility analysis of three recombinant proteins**

The expression and solubility of eGFP (panel A), MMP13 (panel B) and GDF8 (panel C) and their fusion variants were analyzed by SDS-PAGE. For eGFP, four fusion tags including Iasp (I-eGFP, 33.1 kDa), Trx (T-eGFP, 40.8 kDa), NusA (N-eGFP, 83.9 kDa) and MBP (M-eGFP, 69.3 kDa) were tested. For MMP13 (M13, 27.9 kDa) and GDF8 (12.5 kDa), only Iasp (I-MMP13, 33.2 kDa; I-GDF8, 17.8 kDa) and Trx (T-MMP13, 41.8 kDa; T-GDF8, 26.4 kDa) tags were compared. For each sample, the total proteins (T), the soluble component after cell lysis (S) and the insoluble part (IS) were loaded, respectively. Lane “-” is the negative controls sampled from the BL28aD cell culture. Lane “M” is the molecular weight standards and lane “L” is the LE buffer containing 30 U/μL lysozyme. The arrows indicate target bands of these proteins.


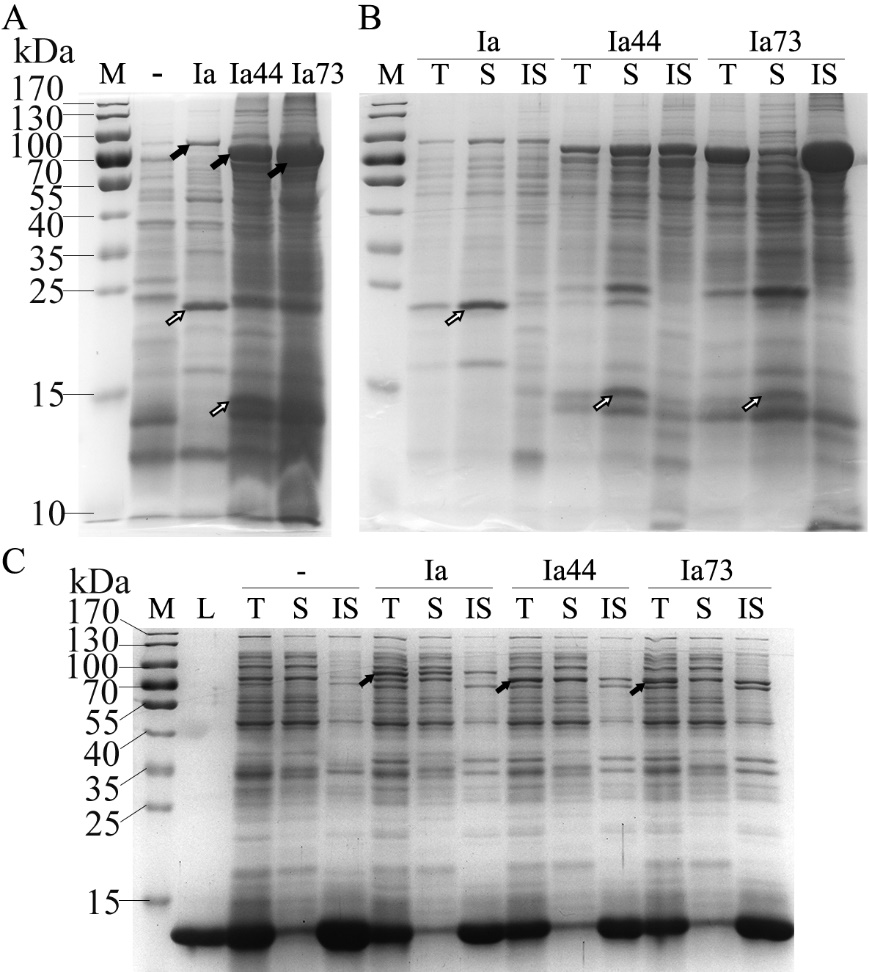


**Figure S3 SDS-PAGE analysis of Cry1Ia and its truncated variants**

**A:** The expression of Cry1a (Ia, 81.2 kDa) and its N-terminal truncated variant Cry1IaD44 (Ia44, 76.2 kDa) and Cry1IaD73 (Ia73, 73.3 kDa) in Bt strain were analyzed by SDS-PAGE. The PBS buffer resuspended cells were sampled and loaded into the 10% gel. Lane “-” is the negative controls sampled from the B304 strain and lane “M” is the molecular weight standards. The black arrows indicate expression products of the three proteins and the hollow arrows indicate the mainly degraded band of Cry1Ia and Cry1IaD44 proteins.

**B:** The solubility analysis of Cry1Ia (Ia), Cry1IaD44 (Ia44) and Cry1IaD73 (Ia73) produced in Bt cells. For each sample, the total proteins (T), the soluble (S) and insoluble component (IS) after alkali treatment (50mM Na_2_CO_3_, 10 mM dithiothreitol, pH 10.5, 37℃ for 1hour) were compared. The soluble and insoluble samples were concentrated 5 times before loading. Lane “M” is the molecular weight standards.

**C:** The expression and solubility of Cry1Ia (Ia), Cry1IaD44 (Ia44) and Cry1IaD73 (Ia73) in *E. coli* BL21-star (DE3) were analyzed by SDS-PAGE. For each sample, the total proteins (T), the soluble component after cell lysis (S) and the insoluble part (IS) were loaded, respectively. “-” is the negative controls sampled from the BL28aD cell culture. Lane “M” is the molecular weight standards and lane “L” is the LE buffer containing 30 U/μL lysozyme. The black arrows indicate target bands of the three proteins.
